# It's Sad but I Like It: The Neural Dissociation Between Musical Emotions and Liking in Experts and Laypersons

**DOI:** 10.3389/fnhum.2015.00676

**Published:** 2016-01-06

**Authors:** Elvira Brattico, Brigitte Bogert, Vinoo Alluri, Mari Tervaniemi, Tuomas Eerola, Thomas Jacobsen

**Affiliations:** ^1^Center for Music in the Brain (MIB), Department of Clinical Medicine, Aarhus University and Royal Academy of Music Aarhus/Aalborg (RAMA)Aarhus, Denmark; ^2^Cognitive Brain Research Unit, Institute of Behavioural Sciences, University of HelsinkiHelsinki, Finland; ^3^Advanced Magnetic Imaging Centre, Aalto UniversityEspoo, Finland; ^4^Department of Music, University of JyväskyläJyväskylä, Finland; ^5^Neuroscience of Emotion and Affective Dynamics Lab, University of GeneveGeneve, Switzerland; ^6^Cicero Learning, University of HelsinkiHelsinki, Finland; ^7^Department of Music, Durham UniversityDurham, UK; ^8^Experimental Psychology Unit, Helmut Schmidt University/University of the Federal Armed Forces HamburgHamburg, Germany

**Keywords:** music, emotion perception, aesthetics, liking, fMRI, salience network, limbic system

## Abstract

Emotion-related areas of the brain, such as the medial frontal cortices, amygdala, and striatum, are activated during listening to sad or happy music as well as during listening to pleasurable music. Indeed, in music, like in other arts, sad and happy emotions might co-exist and be distinct from emotions of pleasure or enjoyment. Here we aimed at discerning the neural correlates of sadness or happiness in music as opposed those related to musical enjoyment. We further investigated whether musical expertise modulates the neural activity during affective listening of music. To these aims, 13 musicians and 16 non-musicians brought to the lab their most liked and disliked musical pieces with a happy and sad connotation. Based on a listening test, we selected the most representative 18 sec excerpts of the emotions of interest for each individual participant. Functional magnetic resonance imaging (fMRI) recordings were obtained while subjects listened to and rated the excerpts. The cortico-thalamo-striatal reward circuit and motor areas were more active during liked than disliked music, whereas only the auditory cortex and the right amygdala were more active for disliked over liked music. These results discern the brain structures responsible for the perception of sad and happy emotions in music from those related to musical enjoyment. We also obtained novel evidence for functional differences in the limbic system associated with musical expertise, by showing enhanced liking-related activity in fronto-insular and cingulate areas in musicians.

## Introduction

Music can convey emotions in a relatively systematic manner within a given music (sub)culture. Adult listeners as well as school-age children are able to perceive and recognize basic emotions expressed by music, particularly happiness and sadness (Krumhansl, 1997; Peretz et al., 1998; Juslin and Laukka, 2004; Baumgartner et al., 2006; Koelsch, 2010; Nieminen et al., 2012). Complex emotions such as love, pride, and jealousy are instead less reproducible by music (Juslin and Laukka, 2004). Basic emotions are characterized by their adaptive or utilitarian function for the behavioral adjustment of individuals to events (Ekman, 1999). Consequently, the basic emotions induced by music do not coincide with the basic emotions triggered by prototypical life events (loss, threat, etc.; Ekman, 1999). It has been suggested that basic emotions experienced in a musical context are weaker than the same emotions occurring in real life and lack the action tendencies typical of basic emotions as well as the “universal” physiological reactions reproduced in individuals of any age and from different cultural background (Scherer, 2004; Zentner et al., 2008). Recent neuroimaging studies aimed at finding the neural correlates of basic emotions in music highlighted the role of the auditory cortex in the superior temporal gyrus, the cingulate cortex, the precuneus, and structures belonging to the reward circuit and limbic system, such as the ventral striatum and the insula, for the perception and induction of happy, joyful music (when compared with neutral or sad music; Mitterschiffthaler et al., 2007; Brattico et al., 2011; Park et al., 2013). Music perceived as sad (compared to neutral or happy music) has also been associated with neural activity in the superior temporal gyrus, the cingulate cortex, the hippocampus/amygdala, and with paralimbic and reward structures such as the ventromedial prefrontal cortex, caudate, and thalamus (Khalfa et al., 2005; Mitterschiffthaler et al., 2007; Brattico et al., 2011). In sum, studies searching for neural correlates of sadness and happiness in music consistently highlighted the role of medial frontal cortices, amygdala, and striatum in generating these emotions.

Along with basic emotions, music induces a separate set of emotions (sometimes termed aesthetic emotions) that are accompanied by evaluative judgments based on formal properties, such as beauty or performance mastering (Scherer, 2004; Silvia, 2005; Brattico and Pearce, 2013; Brattico et al., 2013). Aesthetic emotions in music are typically positive and, following Konecni's (2008) proposal, may be classified as the emotion of “being moved,” the sensation of “thrill” or the sublime awe for a beautiful stimulus. Most scholars have, however, focused on the aesthetic emotion of enjoyment or pleasure derived from a musical activity, which, when conscious, leads to the evaluative judgment of liking a musical piece (Konecni, 2008; Brattico et al., 2013). Musical pleasure has been studied in several ways in the neuroimaging and psychophysiology literature: either by asking subjects to bring their favorite music that induces chills in them (shivers down the spine and goose bumps; Blood and Zatorre, 2001; Grewe et al., 2009), by contrasting classical, instrumental music clips with acoustically-balanced counterparts (Menon and Levitin, 2005; Koelsch et al., 2006) or by correlating behavioral measures of pleasantness (valence) and arousal with brain recordings (Mikutta et al., 2012, 2014; Altenmüller et al., 2014; Jäncke et al., 2015; Trost et al., 2015). These studies showed the involvement of ventromedial and orbitofrontal cortices, amygdala, insula, regions of the reward circuit (particularly, ventral tegmental area and nucleus accumbens), dorsomedial frontal motor areas, hippocampus, anterior cingulate cortex, auditory cortices, and temporal pole during felt musical pleasure or perceived positive valence in music (Blood et al., 1999; Blood and Zatorre, 2001; Brown et al., 2004; Menon and Levitin, 2005; Koelsch et al., 2006; Flores-Gutiérrez et al., 2007; Gosselin et al., 2007; Salimpoor et al., 2013; Trost et al., 2015; Alluri et al., in press). On the other hand, unpleasantness or negative valence from music listening activates the parahippocampal gyrus, amygdala and hippocampus, temporal pole, left anterior cingulate and post-central gyrus, and insula (Blood et al., 1999; Pallesen et al., 2005; Koelsch et al., 2006; Flores-Gutiérrez et al., 2007; Suzuki et al., 2008; Trost et al., 2015). From this brief overview, it is evident that several brain correlates of musical pleasure and displeasure overlap with those for sad and happy emotions in music (for a meta-analysis of brain structures associated with music emotions, cf. Koelsch, 2014).

Psychologically, the overlap or mixture of aesthetic enjoyment and discrete, even negative, emotions, is exemplified by the “tragedy paradox,” a fascinating paradox in music and other arts (Schubert, 1996; Evans and Schubert, 2008). Behavioral studies repetitively showed that the perception of negative basic emotions in music does not correspond with the induction of negative emotions (Juslin and Laukka, 2004; for a review, see Sachs et al., 2015). For instance, a negatively valenced musical passage (such as the Albinoni's Adagio) may be liked by listeners and hence may induce the positive aesthetic emotion of enjoyment. In other words, tears and joy might co-occur during music listening (Vuoskoski and Eerola, 2012; Vuoskoski et al., 2012; Garrido and Schubert, 2013; Taruffi and Koelsch, 2014). Recently, music psychologists observed that people with depression or specific personality traits, such as openness to experience and nostalgia-proneness, possess a greater tendency to prefer listening to sad music. Empathy (the capacity to experience emotions that match those of another person) and absorption (the ability to concentrate so much that awareness of the passage of time and of the external surroundings are lessened) have been found as strongly predictive of liking sad music (Vuoskoski and Eerola, 2011). In relation to these findings, a dissociation theory of aesthetic enjoyment has been proposed, according to which listeners with a propensity for absorption are able to dissociate or de-activate displeasure feelings in aesthetic context, allowing the enjoyment of the emotionally intense stimuli while disregarding their negative content (Garrido and Schubert, 2013). Other authors have argued for an even bolder explanation, such that sad music directly induces pleasant emotions by virtue of the vicarious nature of the musical artistic stimulus (Kawakami et al., 2013, 2014). The reasons to listen to sad music identified in an online survey study (Van den Tol and Edwards, 2013) were the connection with the musical piece or its lyrics, the message communicated, and a high aesthetic value of the music. In sum, the confounding co-presence of emotions during music listening might be closely linked to the overlapping neural activations obtained in the neuroimaging studies described above.

Furthermore, while much is known about how long-term musical training shapes the auditory and somatomotor brain functions and related structures (Fauvel et al., 2014; Pantev et al., 2015; Reybrouck and Brattico, 2015; Schlaug, 2015), little is known concerning its role on emotional musical experience and on the associated brain mechanisms. Preliminary evidence on differences in limbic system functions associated with musical expertise was obtained by James et al. (2008): electric neural activity originating from right medial-temporal structures, including the insula, amygdala, and hippocampal complex, was registered selectively in musicians during listening to chord incongruities inserted in expressive music. Furthermore, an enhanced reactivity of the auditory cortex to unpleasant chords in musicians has been noticed in a neurophysiological study (Brattico et al., 2009). Another neurophysiological study (Mikutta et al., 2014) found enhanced slow mid-frontal theta band activity in professional classical musicians from Austria as opposed to amateur musicians (playing an instrument as hobby) during continuous listening to the first movement of the 5th Symphony by L. van Beethoven. This effect was positively correlated with ratings of pleasantness (valence) of the music, obtained in a session subsequent to the brain recordings (cf. also Mikutta et al., 2012). Musicians (like actors) are often exposed to emotional sounds and, in addition, are trained to express emotions through their playing (Brown et al., 2015). Also, their very reason for starting to play or for choosing music as a profession can often be traced back to their emotional connection to music (Sloboda, 1992). The success of a musician's interpretation and communication of the music (whether from classical, pop/rock or any other genre) relies on her capacity to convey and induce emotions in the listeners (Brown et al., 2015). In other sensory domains, the continuous exposure to a specific set of emotional stimuli alters the neural responses to them in the limbic and reward structures (Kirk et al., 2009). For instance, the bilateral orbitofrontal cortex and the subcallosal anterior cingulate were more active during aesthetic judgments of buildings in a group of architects as compared to controls, even in the absence of group differences in behavioral aesthetic ratings. Recent studies provided initial evidence that the activity and connectivity of the nucleus accumbens in the ventral striatum is enhanced in musicians as compared with non-musicians while listening to expressive or pleasurable (vs. inexpressive or non-pleasurable) music (Chapin et al., 2010; Alluri et al., in press). Based on these findings, it is plausible to hypothesize changes in limbic functions in musicians who have listened to and produced emotionally loaded musical sounds for several years.

Here we wished to disentangle the neural correlates of perception and induction of basic emotions and felt enjoyment (exemplified by liking or disliking) of the same musical material. We additionally examined the effects of musical expertise on this neural relationship. To this end, we asked subjects to bring four of their most liked and disliked musical pieces of happy or sad content to the laboratory. From those pieces, we extracted 18 sec samples and on the basis of a listening test where subjects rated the pieces along several affective scales, we selected the most representative samples for the fMRI session. This was complemented by a listening test, which served to obtain a fine affective classification of the music by subjects and by a detailed acoustic analysis of the music, which instead aimed at extracting the acoustic parameters that might co-vary with the behavioral and brain responses. Perceiving basic emotions in music was expected to involve dissociable limbic and paralimbic brain structures differentially associated with the control of happy and sad emotions, such as the amygdala (Gosselin et al., 2007), the anterior cingulate cortex and insula (Damasio et al., 2000; Lindquist et al., 2012), and the orbitofrontal cortex (Kringelbach, 2005). Furthermore, liking of music should activate the reward system, and in particular the nucleus accumbens, the ventral caudate and the ventral tegmental area (Berridge and Kringelbach, 2008), as previously observed in association with “music chills” and listening to pleasant unfamiliar and familiar music (Blood and Zatorre, 2001; Pereira et al., 2011; Salimpoor et al., 2011, 2013; Zatorre and Salimpoor, 2013). Disliked musical excerpts might also activate areas related to processing of musical dissonance or unpleasantness, such as the parahippocampal gyrus and the temporal poles (Blood et al., 1999; Koelsch et al., 2006).

## Method

### Subjects

Twenty-nine subjects without any neurological, hearing, or psychological disorder participated in the study (15 females; mean age 23.9 ± 3.1 SD). All subjects were chosen from the age group from 18 to 27 years old, as this has been defined as the age when individuals form their musical taste and have the strongest musical experiences (LeBlanc et al., 1996). Sixteen subjects (mean age: 25.1 ± 2.4 SD; 8 females) were classified as non-musicians since they did not receive any formal musical education apart for few years during their childhood and did not play music professionally (earning money from performance). Some of them could be considered, though, as music amateurs since they had played an instrument as hobby (5 had taken lessons in guitar, 8 in piano and 6 had participated at choirs). Out of the non-musicians only 3 had never played an instrument, whereas 7 had tried to learn more than two instruments in their life. Thirteen subjects (mean age: 22.8 ± 3.7 SD; 7 females) declared to be musicians, and indeed possessed long-term formal musical training. Six were educated in and mainly performed classical music, two musicians were trained in and performed folk and jazz music, and the rest played mainly pop/rock music. Five musicians played string instruments, three percussion instruments, two wind instruments, two keyboard instruments, and one was a singer. All musicians, except one, were also able to play other instruments along with their main one. Seven musicians played mainly classical music, whereas the others performed and practiced mainly jazz, folk or rock/pop music. The musicians started to play their main instrument on average at 9.1 ± 3.4 SD years of age and their second instrument at 10.5 ± 3.7 SD years, collecting a total amount of years of training equal, on average, to 16.2 ± 6 SD. Moreover, they reported practicing their instrument on average for 2.2 ± 1.3 SD hours per day at the time of the experiment and to actively listen to music 18.6 ± 15.6 SD hours per week. Non-musicians declared listening to music for 7.6 ± 5.6 SD hours per week at the time of the experiment.

Subjects were recruited online. The recruitment email specified that we were searching for individuals aged 18–27 years old, without a formal musical background or active in music performance but nevertheless with an interest and constancy in listening to music. Moreover, we asked that subjects like music in general, that they also like sad or nostalgic music, and that they listen to music from several genres. Finally, subjects were requested to be healthy and without metal in their bodies. In the recruitment email it was specified that the study would consist of a first session comprising a listening test and a second session an fMRI measurement, and that the participants would receive monetary compensation. The study procedure was approved by the ethical committee of the Helsinki University Hospital and complied with the Helsinki Declaration.

### Procedures

#### Prior to the experiment

Prior to the listening test, subjects were asked to send or bring us 16 music pieces chosen according to the following guidelines: four liked and happy pieces, four liked and sad pieces, four disliked and happy pieces, and four disliked and sad pieces. It was instructed that the pieces should be from different musical genres and that they should not be associated with any special personal memories. The first instruction was meant to increase the acoustic variability of the musical excerpts and hence minimize the correlations between certain sound features and emotional responses to them whereas the second instruction was aimed at avoiding the possible confound of memory associations external to the music and their effects on affective responses. All subjects were able to select the required pieces. Some subjects needed further instructions to select the disliked but familiar pieces, in which case we encouraged them to think of tunes that they casually hear repeatedly from the media. One subject reported in the post-experimental questionnaire not having being able to select pieces without any autobiographical memory associations. Upon a check of the data, and after noticing no striking discrepancies between the other subjects, we opted to keep the data in the sample. The participants either brought the music pieces to the investigator or sent them via an online form. Four excerpts (18 sec each) with 500 ms fade-ins and fade-outs were created from each music piece with Adobe Audition. The majority of the music pieces were pop/rock songs with clear structure (often including verse, chorus, bridge, hook, and refrain; Davidson and Heartwood, 1997), lasting around 3 min each. The four excerpts were taken mainly from the verse, chorus, bridge and refrain. In the case of classical music or other genres not following any common pop form the excerpts were taken from the different parts of the piece to represent the main motifs. Thus, altogether 64 excerpts were cut from the music selection of each individual participant. The loudness level of the excerpts was normalized to a level of −15 dB. The music excerpts were presented binaurally via headphones with Presentation (Neurobehavioral Systems, Ltd., Berkeley, CA).

#### Listening test

To control the reliability of subjects' choices of musical pieces and to ensure that our selection of the 18 sec excerpts complied with subjects' affective categorization, we conducted a listening test at the Cognitive Brain Research Unit, University of Helsinki (approved by the local ethical committee). Each subject performed the test individually on the excerpts extracted from their own self-selected music. Beforehand, the participants filled in a consent form, a questionnaire concerning their musical background and music education especially designed for this study, and the “Music in Mood Regulation” (MMR) questionnaire (Saarikallio, 2008) assessing their use of music-related mood-regulation strategies in their everyday life (the results concerning the MMR questionnaire are presented in two separate papers: Saarikallio et al., 2013; Carlson et al., 2015). Also a questionnaire on musical choices was administered, in which subjects were requested to attribute a musical genre to each of the pieces brought to the lab, and to list reasons for liking or disliking those pieces. Subsequently, the 18 sec music excerpts were delivered in random order with Presentation (Neurobehavioral Systems, Ltd., Berkeley, CA) to the subjects binaurally via headphones at 40 dB above their individually determined hearing thresholds.

By pressing a number from 1 to 5 on a keyboard, subjects rated each excerpt after hearing it according to six 5-step bipolar scales: unfamiliar-familiar, sad-happy, feels sad-feels happy, disliked-liked, unpleasant-pleasant, and ugly-beautiful. In fixed order, the first scale appeared on a screen and when an answer was given, the next scale was presented. Thus, the participants were able to think about their answers for as long as they wanted (the written instructions were as follows: “You will give several ratings on the musical excerpts extracted from the musical pieces you selected after listening to each excerpt. Please follow this procedure. First read the text below and try to memorize the content of each rating. Then listen to the musical except twice. Try to give your ratings on the musical excerpt only without thinking too much about the musical piece to which it belongs”). After the six scales were completed, the next excerpt started by pressing a button. The participants were instructed to rate the excerpts according to the views and feelings they had exactly in that moment. The listening test lasted around 1.5 hour in total.

#### fMRI experiment

The fMRI measurements were conducted with the 3-Tesla scanner (3.0 T Signa VH/I General Electric) in the Advanced Magnetic Imaging (AMI) Centre in the Helsinki University of Technology and were approved by the Coordinating (“Koordinoiva”) ethical committee of the Uusimaa Hospital District and the research committee of the AMI Centre. Before the fMRI measurement, volunteers were informed about the study protocol, signed a written consent form, filled in a safety questionnaire, were encouraged to remove any ferromagnetic material before entering the magnet bore and to relax when in the magnet bore while concentrating on the musical stimuli. During the fMRI session, 33 oblique slices covering the whole brain (field of view 200 × 200 mm; 64 × 64 matrix; slice thickness 4 mm; gap 0 mm) were acquired using an interleaved gradient echo-planar imaging (EPI) sequence (TR = 3 sec; echo time, 32 ms; flip angle 90°) sensitive to blood oxygenation level-dependent (BOLD) contrasts. Continuous acquisition with time to repeat (TR) of 3 sec was used to measure brain responses to the experimental stimuli, assuming that the effect of the scanner noise would be constant and thus easily discernable from the effects of the musical stimulation. Subsequent to a short break after the fMRI session, anatomical T1 weighted MR images (field of view 260 × 260 mm; 256 × 256 matrix; thickness 1 mm; spacing 0 mm) were acquired. The subjects received two movie theater tickets to compensate for their inconvenience after the experiment.

**Figure 1:**
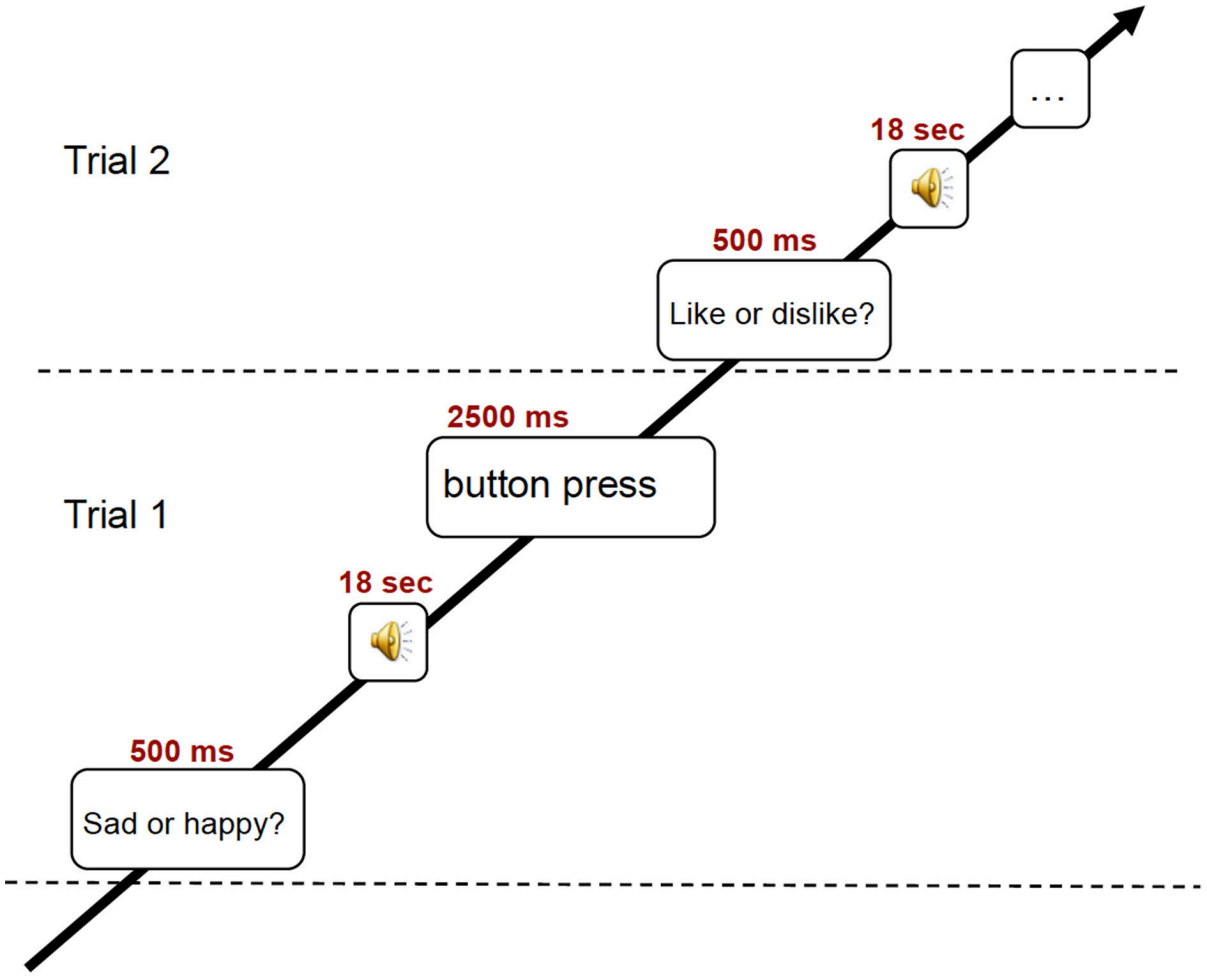
**Schematic illustration of the experimental trial used in the fMRI scanning**.

During the fMRI session, subjects listened to 18 sec excerpts of music selected on the basis of the previously conducted listening test. In detail, from the four excerpts for each of the 16 pieces of music brought to the lab by the subjects, the two excerpts obtaining the highest scores in emotional and in familiarity ratings were fed to the stimulation computer and delivered to the subjects in random order via high-fidelity MR-compatible headphones. The sound level was adjusted to be comfortable at an energy level around 80 dB. In the fMRI scanner, the subjects performed one out of two behavioral tasks, preceded by a visual cue (for an illustration of the experimental paradigm, see Figure 1). In one behavioral task prompted by the text “Like? Dislike?” (in Finnish: “Pidän? En pidä?”), the subjects had to indicate whether they liked the piece or not. In the other behavioral task prompted by the text “Sad? Happy?” (in Finnish: “Surullinen? Iloinen?”), the subjects rated the emotional content of the music on a binary scale. Three test trials were presented to the subjects prior to the main session. The text with the visual cue was maintained for the duration of the stimulus and served as fixation point. At the end of the 18 sec stimulus, another text appeared, asking the subjects to answer to the previously seen question (in Finnish: “Vasta nyt”). For the behavioral answer, subjects pressed with the second and third fingers of left or right hand (counterbalanced between subjects) MR-compatible button pads. After a 3 sec interval without any stimulus, a sinusoidal tone indicated the start of the next trial. The total time of the fMRI session was 21 min. Subsequent to a short break after fMRI recording, anatomical T1 weighted MR images were also acquired in about 10 min.

### Data analysis

#### Acoustic parameters

To explore any possible commonality in the acoustic features contained in liked vs. disliked music and in the happy vs. sad music we conducted two generalized linear mixed models with the participant as repeated measures variable. The familiarity ratings for each musical excerpt were included as covariates, similarly to the fMRI analysis. As dependent variables of the linear mixed model, we entered the mean values for each excerpt resulting from the computational analysis of the musical excerpts conducted with the MIRToolbox version 1.3.3 (developed at the University of Jyväskylä; Lartillot and Toiviainen, 2007). The first 24 different acoustic features were extracted, chosen among the ones most studied in the psychoacoustic literature and having clear perceptual attributes. The features were extracted from the stimulus on a frame-by-frame basis (for more details, see Alluri and Toiviainen, 2010; Alluri et al., 2012). A window length of 25 ms with a 50% overlap was used to extract the timbral features, and a frame size of 2 sec with a 33% overlap was used to extract the tonal and rhythmic features. All the features are documented in the original paper presenting the MIRToolbox and in subsequent studies including the more recently included features (Alluri et al., 2012; Eerola et al., 2012). To minimize Type I errors resulting from multiple comparisons we grouped features into six sets according to a classification as suggested by Eerola (2011), excluding structural features, which were not extracted: Dynamics (root mean square energy, low energy), Rhythm (fluctuation peak, fluctuation centroid, tempo, pulse clarity), Timbre (zero crossing rate, centroid, brightness, skewness, kurtosis, flatness, spectral entropy, roughness, irregularity, spectral flux), Pitch (chroma peak), Tonality (key clarity, mode, HCDF, spectral entropy extracting using a 5 sec frame), Articulation (attach time, attack slope). The six feature classes were represented by six principal component scores explaining 70% variance [*X*^2^ = 1522.7, *p* < 0.001, mean squared error, RMSR = 0.05] in the acoustic features across all tracks submitted by the participants. The statistical analyses concerning acoustic features were conducted in IBM SPSS Statistics version 22.

#### Behavioral ratings in the listening test

The effects of musical expertise on the six different scales of judgments collected in the listening test (familiarity, emotion recognition, emotion induction, liking, pleasantness, and beauty) were investigated in IBM SPSS Statistics version 22, using separate Kruskal-Wallis tests, corresponding to non-parametric mixed ANOVAs with Group as the between-subject factor and the discrete 5-point ratings as the dependent variable. The effects of liking or emotions of the musical stimuli on the six ratings scales were instead studied with separate non-parametric Friedman's rank tests. Pairwise comparisons aiming to test differences between ratings to liked vs. disliked music and happy vs. sad music in musicians and non-musicians were carried out with the non-parametric Wilconxon statistics. Reliability analyses explored the internal consistency, correlation, and covariance of the related scales. Emotion recognition and emotion induction were compared, and so were preference, pleasantness, and beauty, as they are all aesthetic measures.

#### fMRI data

Whole-brain image analysis was completed using Statistical Parametric Mapping 5 (SPM5) and Voxel Morphometry Mapping (VBM) for preprocessing and upgraded to SPM8 for statistical analyses (http://www.fil.ion.ucl.ac.uk/spm). Images for each participant were realigned to adjust for movement between volumes, and then segmented with VBM into gray matter, cerebrospinal fluid and white matter images. The segmented individual images were then spatially normalized onto the Montreal Neurological Institute (MNI) a priori tissue template of the gray matter according to a 12-parameters affine transformation model. The final preprocessing step included spatial smoothing with a Gaussian filter of 6 mm full-width at half maximum (FWHM). The normalization using segmented gray matter images obtained with VBM as an intermediate step was chosen for its superiority over the direct normalization of EPI images to the MNI template according to pilot tests. Smoothed, normalized brain volumes were screened to determine whether they met the criteria for high quality and scan stability as determined by small motion correction (<2 mm translation and <2° rotation). For statistical analysis, the fMRI responses were modeled using a canonical hemodynamic response function (HRF) with time dispersion and temporally filtered using a high-pass filter of 1/128 Hz to minimize scanner drift. The six movement parameters resulting from realignment preprocessing were modeled as regressors of no interest in the analysis.

Following preprocessing, linear contrasts employing canonical HFR function were used to estimate condition-specific blood oxygen level-dependent activation for each individual and each scan. In a first-level analysis, we compared with paired-samples tests the brain responses during the liked stimuli contrasted directly with the brain responses to the disliked stimuli, and vice versa. Moreover, we contrasted with paired-samples tests the brain responses to sad stimuli with the brain responses to the happy stimuli, and vice versa. These individual contrast images (i.e., weighted sum of the beta images) were then used in second-level random effects models that account for both scan-to-scan and participant-to-participant variability to determine mean condition-specific regional responses. General linear models (GLM's) with Group, Liking and Emotion as factors were then performed and *t*-tests were conducted to further investigate the significant main effects and interactions.

Further analyses were conducted to analyze the putative effects of sensory processing on brain responses to musical emotions and liking. To this aim, we chose to conduct region-of-interest (ROI) analysis by extracting the signal change from the clusters of activations found to be significant with Marsbar. We decided to opt for this method, rather than regressing out the acoustic features from the GLM analysis, because of the differential role of acoustic features for discrete emotions and for liking judgments. The signal change values were then entered in IBM SPSS Statistics version 22 for studying correlations with the acoustic feature values of each musical excerpt obtained computationally with MIRToolbox analysis. The alpha level was corrected for multiple comparisons by Bonferroni correction (significance at p < 0.001 when considering only the comparisons for the acoustic features that significantly differentiate the stimulus categories).

For the whole-brain fMRI analyses, a statistical threshold of p < 0.001, minimum cluster size (k) = 29 was used, as resulted from the calculation of the alpha level of significance based on Monte Carlo permutations.

## Results

### Questionnaire on music choices

**Figure 2:**
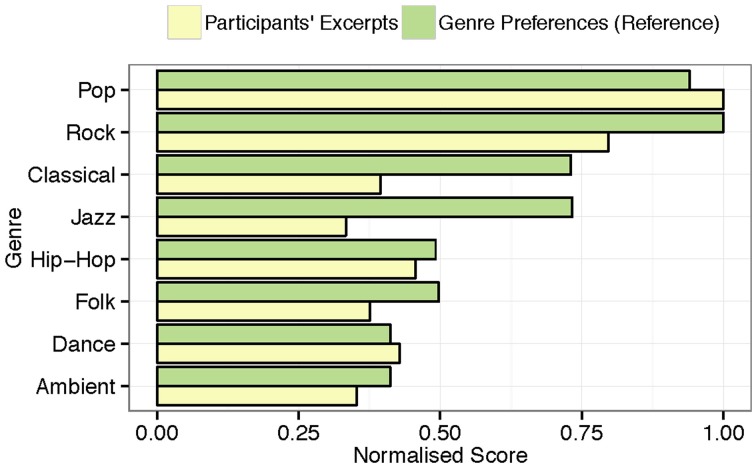
**Normalized distribution of musical genres represented by the musical excerpts brought to the lab by the participants and normalized genre preferences from a comparable sample (***n*** = 346)**.

The subjects classified their self-selected musical pieces as belonging to several different genres, e.g., pop, rock, emo, sugary ballad, Finnish *iskelmä* (melodic pop like “Schlagers”), classical, folk, electronic, and atonal music, etc. They showed detailed knowledge of the musical genres, and their selection reproduces the distribution of musical genre preferences in the Western world, with pop/rock as the most widely listened genre (80%). As illustrated in Figure 2, these findings are analogous to those of a similarly aged (M = 20.64, *SD* = 2.84) reference sample (*n* = 346) from the same country (Ferrer et al., 2013), who also predominantly listen to pop/rock music.

### Behavioral ratings in the listening test

Separate Kruskal-Wallis tests for the six behavioral ratings showed that none of the ratings strongly differed between musicians and non-musicians [main effect of Group for all: $\chi_{\left(1\right)}^{2}<$ 2.2; *p* > 0.14], except for the emotion recognition of disliked sad music [$\chi_{\left(1\right)}^{2}=$ 5.4; *p* = 0.02], with non-musicians classifying the disliked music as sadder (*M* = 1.7 ±.4 SD) than musicians (*M* = 2.0 ±.3 SD). Considering that ratings did not consistently differentiate musicians from non-musicians we conducted the subsequent analyses studying effects of Liking and Emotion factors using the whole group of participants.

The familiarity ratings (familiar vs. unfamiliar) differed between stimuli [$\chi_{\left(3\right)}^{2}=54.9$; *p* < 0.0001] with the liked music rated as overall more familiar than disliked music (*Z* = −3.9 for happy music, and *Z* = −4.2 for sad music, with *p* < 0.0001 for both) and the disliked happy music rated as more familiar than the disliked sad music (*Z* = −2.1, *p* = 0.04). However, for all stimuli the mean scores were positive (>3.9), and hence the musical pieces were overall familiar to subjects.

The significant result for the emotion recognition ratings (happy vs. sad) [$\chi_{\left(3\right)}^{2}=75.9$; *p* < 0.0001] revealed that liked happy music was better recognized as happy than disliked happy (*Z* = −3.3; *p* = 0.001) and the same applied for sad music (*Z* = −3.1; *p* = 0.002). The significant result for the emotion induction ratings (feel happy vs. feel sad) [$\chi_{\left(3\right)}^{2}=$ 69.7; *p* < 0.0001] further evidenced that subjects felt more intensively emotions when listening to liked than disliked happy music (*Z* = −4.6; *p* < −0.0001; liked happy music: *M* = 4.4 ±.4 SD; disliked happy music: *M* = 3.2 ±.6 SD) and to liked than disliked sad music (*Z* = −2.2; *p* = 0.03; liked sad music: *M* = 2.1 ±.6 SD; disliked sad music: *M* = 2.3 ±.7 SD).

The liking ratings (liked vs. disliked), as expected, differed between stimulus categories [$\chi_{\left(3\right)}^{2}=$ 74.4; *p* < 0.0001] with sad music obtaining higher liking ratings than happy music (*Z* = −2.1, *p* < 0.04 in Wilcoxon test) and disliked sad or happy music obtaining lower liking ratings than liked sad or happy music (*Z* = −4.7, *p* < 0.0001). Pleasantness ratings (pleasant vs. unpleasant), which also differed between stimuli [$\chi_{\left(3\right)}^{2}=$ 73.8; *p* < 0.0001], were higher for liked (sad or happy) music than disliked (sad or happy) music (*Z* = −4.7, *p* < 0.0001). The beauty ratings (beautiful vs. ugly) differing between stimulus categories [$\chi_{\left(3\right)}^{2}=$ 72.9; *p* < 0.0001] revealed that liked music was rated as more beautiful than disliked music (*Z* = −4.7; *p* < 0.0001) and sad music was also rated as more beautiful than happy music (*Z* = −4.0; *p* < −0.0001; sad music: *M* = 4.6 ±.4 SD vs. happy music: *M* = 4.0 ±.7 SD).

Recognition/induction reliability for liked happy excerpts had a standardized Cronbach's alpha coefficient of 0.865. For liked sad excerpts, this value was 0.660. For disliked happy, it was 0.518, and for disliked sad it was 0.608. Liked happy emotion recognition and liked happy perception were the only two variables that were significantly correlated (*r* = 0.762). Reliability of beauty ratings as measured with Cronbach's alpha coefficient amounted to 0.887 for liked happy, 0.920 for liked sad, 0.915 for disliked happy, and 0.861 for disliked sad music (see Table 1).

**Table 1:** **Inter-item correlations for the behavioral ratings obtained during the listening tests (above the diagonal) and during fMRI sessions (below the diagonal)**.

	**LHpref**	**LHpleas**	**LHbeaut**	**LSpref**	**LSpleas**	**LSbeaut**	**DHpref**	**DHpleas**	**DHbeaut**	**DSpref**	**DSpleas**	**DSbeaut**
LHpref	–	0.877	0.619	0.713	0.725	0.707	−0.444	−0.418	−0.324	−0.291	−0.407	−0.200
LHpleas	0.805	–	0.675	0.567	0.691	0.605	−0.481	−0.414	−0.342	−0.371	−0.525	−0.332
LHbeaut	0.491	0.613	–	0.403	0.323	0.494	−0.290	−0.142	−0.206	−0.233	−0.327	−0.457
LSpref	0.671	0.566	0.325	–	0.833	0.809	−0.676	−0.536	−0.432	−0.407	−0.256	−0.180
LSpleas	0.547	0.520	0.170	0.755	–	0.736	−0.636	−0.582	−0.476	−0.432	−0.342	−0.182
LSbeaut	0.584	0.545	0.356	0.775	0.716	–	−0.669	−0.618	−0.519	−0.304	−0.315	−0.169
DHpref	−0.399	−0.376	−0.125	−0.574	−0.469	−0.724	–	0.839	0.766	0.532	0.540	0.369
DHpleas	−0.382	−0.339	−0.037	−0.420	−0.376	−0.637	0.751	–	0.737	0.353	0.591	0.141
DHbeaut	−0.369	−0.323	−0.231	−0.433	−0.411	−0.592	0.749	0.588	–	0.415	0.541	0.442
DSpref	−0.212	−0.283	−0.120	−0.303	−0.399	−0.340	0.503	0.351	0.393	–	0.771	0.635
DSpleas	−0.472	−0.461	−0.192	−0.279	−0.300	−0.339	0.469	0.597	0.503	0.755	–	0.617
DSbeaut	−0.129	−0.277	−0.416	−0.108	−0.103	−0.191	0.261	0.097	0.417	0.527	0.419	–

*LH, liked happy stimuli; LS, liked sad; DH, disliked happy; and DS, disliked sad. Pref, preference ratings; pleas, pleasantness; and beaut, beauty*.

We also tested whether the results obtained during the listening test were compatible with those obtained during the fMRI measurement in a separate lab. The ANOVA did not reveal any significant main effect of Experiment (*p* > 0.95) validating the experimental procedure. Cronbach's coefficients of reliability for the ratings obtained during the fMRI session is illustrated in Table 1.

### Acoustic parameters

The musical excerpts liked by musicians differed from those chosen by non-musicians in the acoustic values of Articulation [main effect of Group: *F*_(1, 217)_ = 105.2, *p* < 0.0001], Dynamics [main effect of Group: *F*_(1, 262)_ = 787.2, *p* < 0.0001], and Timbre [main effect of Group: *F*_(1, 204)_ = 205, *p* < 0.0001]. The liked and disliked musical excerpts differed from each other on Pitch [main effect of Liking: *F*_(1, 282)_ = 4.1, *p* = 0.04], Articulation [main effect of Liking: *F*_(1, 293)_ = 4.5, *p* = 0.03], Rhythm [main effect of Liking: *F*_(1, 260)_ = 9.9, *p* = 0.002], Timbre [main effect of Liking: *F*_(1, 424)_ = 66.5, *p* < 0.0001]. The differences in acoustic features between sad and happy musical excerpts were even more remarkable: happy significantly differed from sad music in Articulation [main effect of Emotion: *F*_(1, 304)_ = 30.6, *p* < 0.0001], Pitch [main effect of Emotion: *F*_(1, 256)_ = 14.8, *p* < 0.0001], Rhythm [main effect of Emotion: *F*_(1, 236)_ = 63.9, *p* < 0.0001], Timbre [main effect of Emotion: *F*_(1, 229)_ = 33.6, *p* < 0.0001], Tonality [main effect of Emotion: *F*_(1, 239)_ = 6.6, *p* = 0.01]. The acoustic feature content of happy and sad music also differed between musicians and non-musicians for Articulation [main effect of Group: *F*_(1, 301)_ = 185.4, *p* < 0.0001], Dynamics [main effect of Group: *F*_(1, 514)_ = 514.2, *p* < 0.0001], and Timbre [main effect of Group: *F*_(1, 179)_ = 143.1, *p* < 0.0001].

To summarize, the majority (5/6 feature categories) of the differences between the excerpts were observed between happy and sad emotions. These acoustic differences were consistent with the past research on musical features for different emotional expression (e.g., Eerola, 2011). Also the liked and disliked excerpts showed marked differences. Finally, in feature categories such as the Dynamics and Timbre, the excerpts chosen by musicians and non-musicians varied in a systematic fashion. It is likely that the acoustic differences between musical excerpts depending on musical expertise are related to the musical genres of the excerpts chosen by the two experimental groups, although a full analysis of the genre differences are beyond this investigation.

### fMRI responses

#### Overall ANOVAs

**Figure 3:**
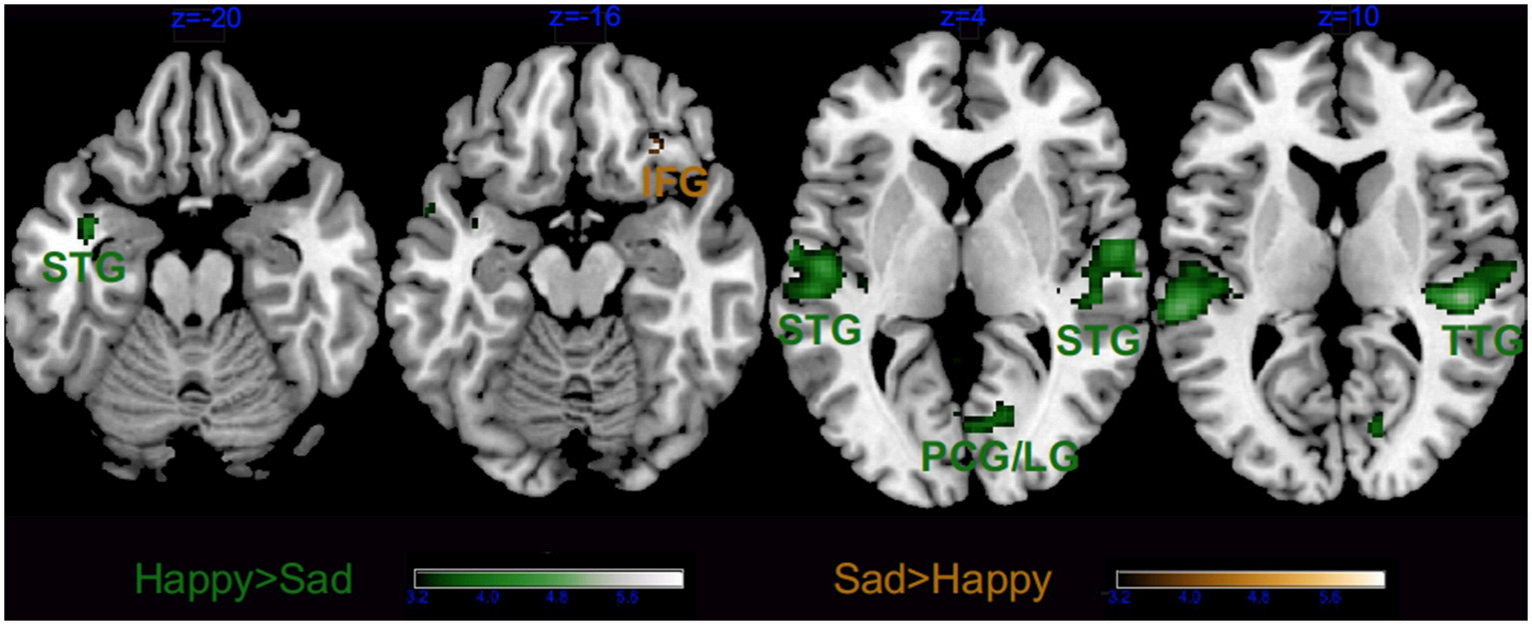
**Main effect of Emotion**. STG, superior temporal gyrus; TTG, transverse temporal gyrus; LG, lingual gyrus; IFG, inferior frontal gyrus and PCG, post-central gyrus. Activations were considered significant at *p* < 0.001, and cluster size *k* > 29 (as obtained with Monte Carlo permutations).

As visible from Figure 3, we obtained a significant main effect of Emotion in several areas, listed in Tables 2, 3, which derived from the higher activity in happy > sad in primary and secondary auditory cortices along the bilateral superior and transverse temporal gyri, and the medial structures such as the cuneus, lingual and posterior cingulate gyri. The contrast sad > happy contributed only with activation in the right lateral prefrontal cortex (inferior frontal gyrus, BA 47/11).

**Table 2:** **Coordinates and statistical values of the full factorial analysis of fMRI responses in both musicians and non-musicians**.

**Region**	**L/R**	**BA**	***x***	***y***	***z***	***Z***	***k***	**Coordinates (x, y, z)**
**Main Effects and Interactions**								
**LIKING**								
Middle/superior temporal gyrus	L	BA 21/22	−59	−8	−6	6.58	1549	−60, −8, −8
Middle/superior temporal gyrus	R	BA 21/38	61	−6	−3	6.53	1524	62, −6, −4
Medial frontal gyrus/anterior cingulate cortex	R/L	BA 6/ 24	6	−3	61	6.16	888	6, −6, 66
Cingulate gyrus/paracentral lobule	R/L	BA 31	8	−27	40	5.51	671	8, −30, 42
Culmen	L	N/A	−34	−58	−24	5.08	112	−34, −58, −32
Culmen/declive	R/L	N/A	6	−57	−19	4.71	380	6, −58, −26
Precuneus	R	BA7	10	−56	47	4.53	50	10, −60, 48
Anterior nucleus	R/L	N/A	6	−3	9	4.37	258	6, −4, 10
Precentral gyrus	L	BA 6	−46	−2	39	4.07	87	−46,−4, 42
Precentral gyrus	L	BA 6	−61	3	13	4.06	79	−62, 2, 14
Caudate body	R	N/A	20	−3	22	4.04	75	20, −4, 24
Caudate body	L	N/A	−18	1	22	3.98	43	−18, 0, 24
Thalamus	L	N/A	0	−19	16	3.98	50	0, −20, 16
Parahippocampal gyrus	R	BA 20	40	−28	−15	3.97	42	40, −28, −20
Superior temporal gyrus	R	BA 22	48	2	4	3.86	57	48, 2, 4
Declive/culmen	R	N/A	28	−63	−22	3.83	117	28, −64, −30
Post-central gyrus	L	BA 3	−28	−34	51	3.80	38	−28, −38, 54
Culmen	R	N/A	6	−34	−12	3.63	32	6, −34, −16
Lingual gyrus	L	BA 18	−22	−72	−1	3.61	31	−22, −74, −6
Middle frontal gyrus	L	BA 6	−24	−1	61	3.60	42	−24, −4, 66
Pulvinar	L/R	N/A	−2	−27	3	3.51	46	−2, −28, 2
**EMOTION**								
Superior temporal gyrus	L	BA 22/ 41	−51	−14	−1	5.15	967	−52, −14, −2
Transverse/superior temporal gyrus	R	BA 41/ 22	46	−25	10	4.75	565	46, −26, 10
Superior temporal gyrus	R	BA 22	50	4	−5	3.90	39	50, 4, −6
Posterior cingulate/lingual gyrus	R	BA 30/18	16	−68	7	3.56	75	16, −70, 4
**GROUP**								
Declive	R	N/A	28	−65	−19	4.49	62	28, −66, −26
Precuneus	L	BA 7	−22	−62	36	4.24	29	−22, −66, 36
Precuneus	R	BA 7	24	−60	38	3.90	39	24, −64, 38
Post-central gyrus	L	BA 3	−20	−30	53	3.61	31	−20, −34, 56
Precentral gyrus	L	BA 6	−44	−12	39	3.52	30	−44, −14, 42
**LIKING × GROUP**								
Cerebellar tonsil	L	N/A	−10	−50	−34	3.70	32	−10, −50, −44
**EMOTION × GROUP**								
Declive	R	N/A	4	−65	−15	4.49	118	4,−66, −22
Red nucleus	L	N/A	−2	−26	−14	4.32	40	−2,−26, −18
Posterior cingulate	R	BA 30	20	−52	15	3.60	32	20, −54, 14

*Clusters were considered significant at p < 0.001, and cluster size k>29 (as obtained with Monte Carlo permutations). Coordinates are in MNI space. Only effects and interactions producing significant clusters are reported*.

**Table 3:** **Coordinates and statistical values of the ***t***-tests on fMRI responses in both musicians and non-musicians**.

***T*****-tests**								
**LIKE >DISLIKE**								
Medial frontal gyrus/anterior cingulate	R/L	BA 6/24	6	−3	61	6.27	1223	6, −6, 66
Cingulate gyrus/paracentral lobule	R/L	BA 31	8	−27	40	5.63	802	8, −30, 42
Culmen/declive	L/R	N/A	−34	−58	−24	5.21	865	−34, −58, −32
Precuneus	R	BA 7	10	−56	47	4.68	63	10, −60, 48
Anterior nucleus (Thalamus)	R/L	N/A	6	−3	9	4.51	497	6, −4, 10
Precentral/post-centralgyrus	L	BA 6/3	−46	−2	39	4.23	169	−46, −4, 42
Precentral gyrus	L	BA 6	−61	3	13	4.22	105	−62, 2, 14
Caudate body	R	N/A	20	−3	22	4.20	114	20, −4, 24
Caudate body/tail	L	N/A	−18	1	22	4.14	99	−18, 0, 24
Parahippocampal gyrus	R	BA 20	40	−28	−15	4.14	55	40, −28, −20
Superior temporal gyrus	R	BA 22	48	2	4	4.03	91	48, 2, 4
Middle frontal gyrus	L	BA 6	−24	8	47	4.02	45	−24, 6, 52
Post-central gyrus	L	BA 3	−28	−34	51	3.96	59	−28, −38, 54
Medial frontal gyrus	R	BA 10	8	54	−9	3.91	42	8, 56, −8
Precuneus	R	BA 7	8	−46	54	3.81	45	8, −50, 56
Culmen	R	N/A	6	−34	−12	3.81	58	6, −34, −16
Culmen	R	N/A	4	−60	−4	3.72	36	4, −62, −8
**DISLIKE >LIKE**								
Middle temporal gyrus	L	BA 21/22	−59	−8	−6	6.69	1719	−60, −8, −8
Middle temporal gyrus	R	BA 21/38	61	−6	−3	6.64	1722	62, −6, −4
Amygdala	R	N/A	22	−7	−15	4.11	30	22, −6, −18
Lingual gyrus	L	BA 18	−22	−72	−1	3.78	53	−22, −74, −6
**HAPPY >SAD**								
Superior temporal gyrus	L	BA 22/41	−51	−14	−1	5.28	1228	−52, −14, −2
Transverse/superior temporal gyrus	R	BA 41/22	46	−25	10	4.89	858	46, −26, 10
Superior temporal gyrus	L	BA 38	−40	−1	−17	4.00	41	−40, 0, −20
Posterior cingulate/lingual Gyrus/cuneus	R/R/L	BA 30/18/30	16	−68	7	3.73	127	16, −70, 4
**SAD >HAPPY**								
Inferior frontal gyrus	R	BA 47/11	30	26	−15	3.70	41	30, 28, −16
**MUSICIANS >NON-MUSICIANS**								
Declive	R	N/A	28	−65	−19	4.63	82	28, −66, −26
Precuneus	L	BA 7	−22	−62	36	4.40	44	−22, −66, 36
Precuneus	R	BA 7	24	−60	38	4.07	65	24, −64, 38
Precentral gyrus	R	BA 6	38	−10	35	3.98	42	38, −12, 38
Cingulate gyrus	L	BA 24	−4	−12	37	3.95	32	−4, −14, 40
Post-central gyrus	L	BA 3	−20	−30	53	3.78	53	−20, −34, 56
Ventral lateral nucleus/mammillary body	L	N/A	−16	−17	10	3.71	34	−16, −18, 10
Precentral gyrus	L	BA 6	−44	−12	39	3.70	73	−44, −14, 42
Insula	L	BA 13	−40	−32	20	3.69	36	−40, −34, 20
Inferior parietal lobule	R	BA 40	46	−48	47	3.67	62	46, −52, 48
**NEGATIVE INTERACTION: LIKING × EMOTION (LS +DH>LH + DS)**								
Parahippocampal gyrus	R	BA 19	30	−47	1	3.74	35	30, −48, −2
**POSITIVE INTERACTION: LIKING × GROUP (LHNM + LSNM + DHM + DSM>LHM + LSM + DHNM + DHM)**								
Cerebellar tonsil	L	N/A	−10	−50	−34	3.87	54	−10, −50, −44
**POSITIVE INTERACTION: EMOTION × GROUP (LHNM + LSM + DHNM + LSM>LHM + LSNM + DHM + LHNM)**								
Declive	R	N/A	4	−65	−15	4.63	212	4, −66,−22
Red nucleus	L	N/A	−2	−26	−14	4.47	57	−2,−26, −18
Posterior cingulate	R	BA 30	20	−52	15	3.78	44	20, −54, 14
Superior temporal gyrus	L	BA 22	−57	2	5	3.48	29	−58, 2, 6
Claustrum/insula	L	BA 13	−36	−11	12	3.45	38	−36, −12, 12
**POSITIVE INTERACTION: LIKING × EMOTION × GROUP (LHNM + LSM+DHM+DSNM>LHM + LSNM +DHNM + DSM)**								
Inferior frontal gyrus	R	BA 47	40	17	−9	3.57	30	40, 18, −10
**NEGATIVE INTERACTION: LIKING × EMOTION × GROUP (LHM + LSNM +DHNM + DSM>LHNM + LSM+DHM+DSNM)**								
Insula/transverse temporal gyrus	L	BA 13/41	−38	−28	16	3.60	40	−38, −30, 16

*Clusters were considered significant at p < 0.001, and cluster size k>29 (as obtained with Monte Carlo permutations). Coordinates are in MNI space. Only effects and interactions producing significant clusters are reported*.

**Figure 4:**
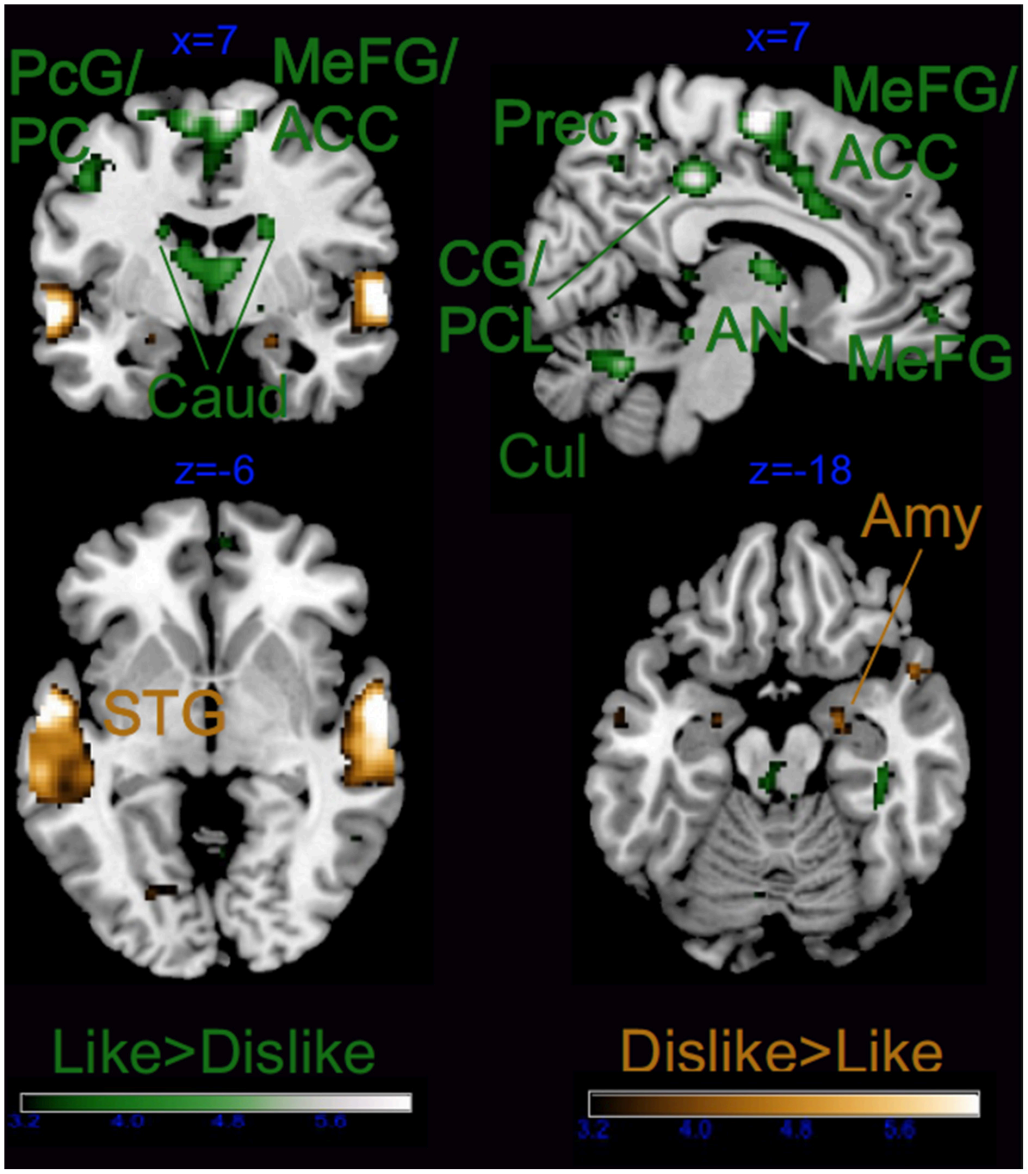
**Main effect of Liking**. ACC, anterior cingulate cortex; Caud, caudate; CG, cingulate gyrus; PCL, paracentral lobule; PC, posterior cingulate; Cul, cerebellar culmen; STG, superior temporal gyrus; Amy, amygdala; AN, thalamic anterior nucleus; Prec, precuneus; and PcG, precentral gyrus. Activations were considered significant at *p* < 0.001, and cluster size *k*> 29 (as obtained with Monte Carlo permutations).

As illustrated in Tables 2, 3 and Figure 4, most of the activations obtained by the Liking factor in the GLM were driven by the contrast Liked > Disliked. Listening to liked music over disliked music activated large clusters in the bilateral medial frontal, anterior cingulate gyri, and paracentral lobule in addition to the bilateral caudate nucleus of the basal ganglia, and the anterior nucleus of the thalamus. In the right hemisphere, we found activations in the parahippocampal gyrus, the superior temporal gyrus, the precuneus and the medial frontal gyrus whereas activations of somatomotor areas (precentral, post-central, and middle frontal gyri) were lateralized to the left hemisphere. Several loci were activated in the cerebellum, including bilaterally the culmen and declive. On the other hand, the opposite contrast Disliked > Liked music resulted in activations only in the bilateral middle temporal gyri, the right amygdala and the left lingual gyrus.

**Figure 5:**
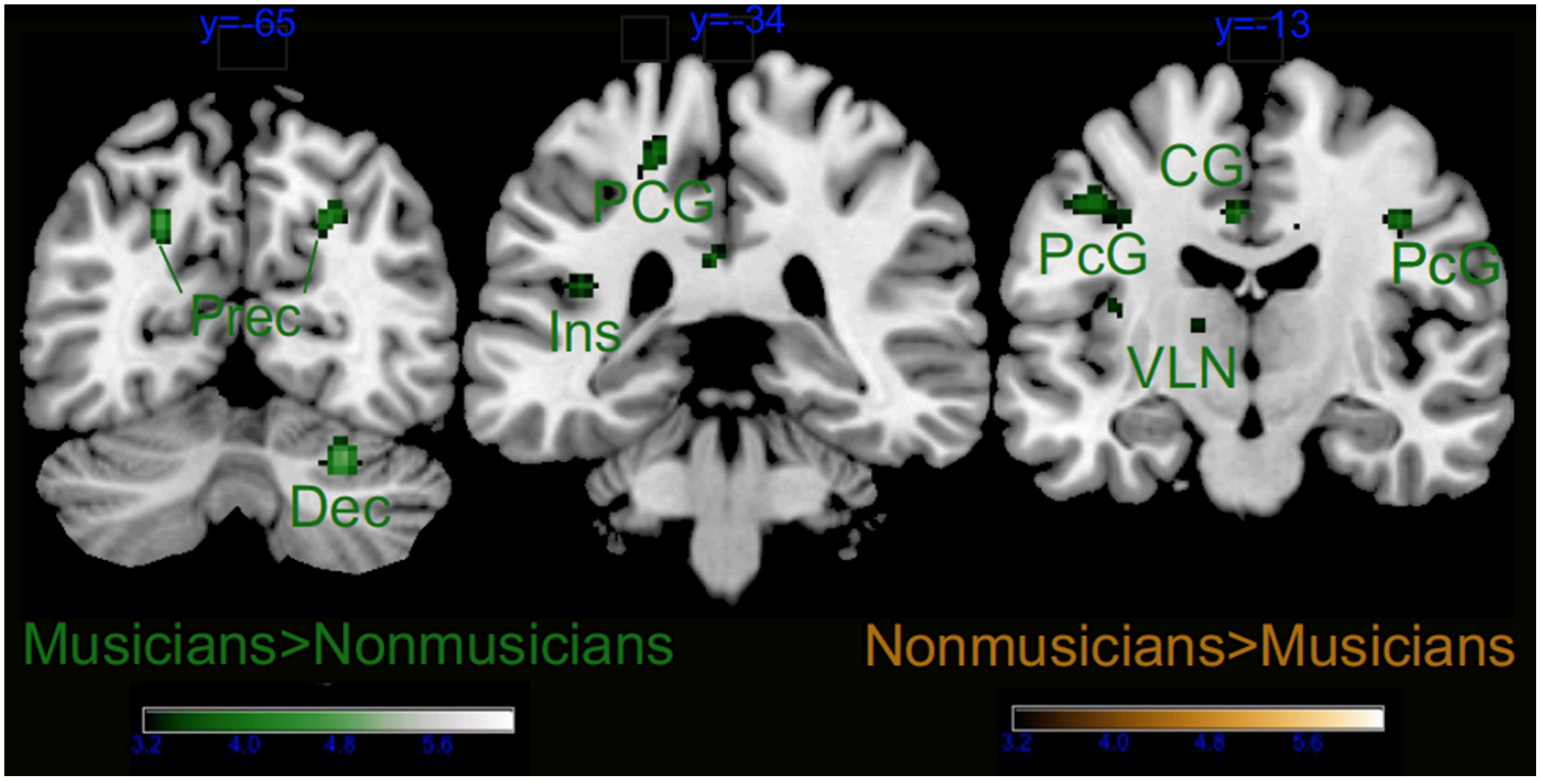
**Main effect of Group**. Dec, cerebellar declive; Ins, insula; PcG, precentral gyrus; PCG, post-central gyrus; CG, cingulate gyrus; and VLN, ventral lateral nucleus of the thalamus. Activations were considered significant at *p* < 0.001, and cluster size *k* > 29 (as obtained with Monte Carlo permutations).

Group as factor also activated several brain areas alone or in interaction with the other factors (see Tables 2, 3 and Figure 5). As revealed by *t*-tests for the main effect of Group, the main differences between musicians and non-musicians were obtained in somatomotor regions such as the left post-central and precentral gyri, and the right declive of the cerebellum and were explained by their larger activity in the musicians' brains. On the other hand, the non-musicians' brains were never more active than that of musicians during affective listening to music.

We also obtained significant clusters of activation for the interaction of Group × Emotion in the right posterior cingulate, the red nucleus of the left brainstem, and the right declive of the cerebellum. Liking × Group also was significant in the tonsil of the cerebellum whereas the 3-way interaction Group × Liking × Emotion did not yield any significant activation at our Monte Carlo based alpha level, but when allowing for smaller cluster size, interaction in the brain activity was observed in the insula and the anterior cingulate cortex, as reported in the following sections. To study more closely the complex 3-way interaction of Group × Liking × Emotion we conducted separate analyses for musicians and non-musicians.

#### Separate GLM for musicians

**Figure 6:**
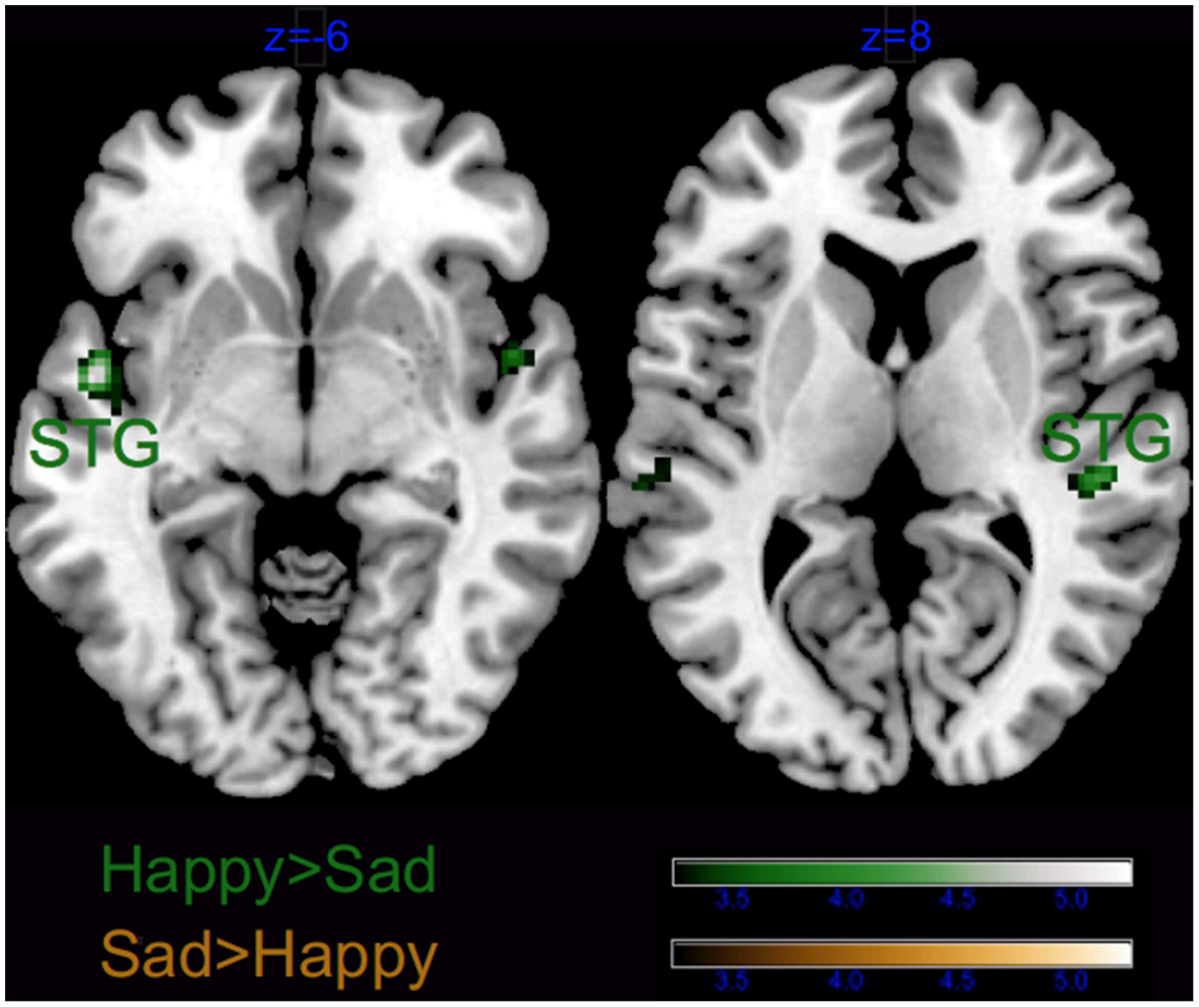
**Main effect of Emotion for musicians only**. STG, superior temporal gyrus. Activations were considered significant at *p* < 0.001, and cluster size *k*> 29 (as obtained with Monte Carlo permutations).

**Figure 7:**
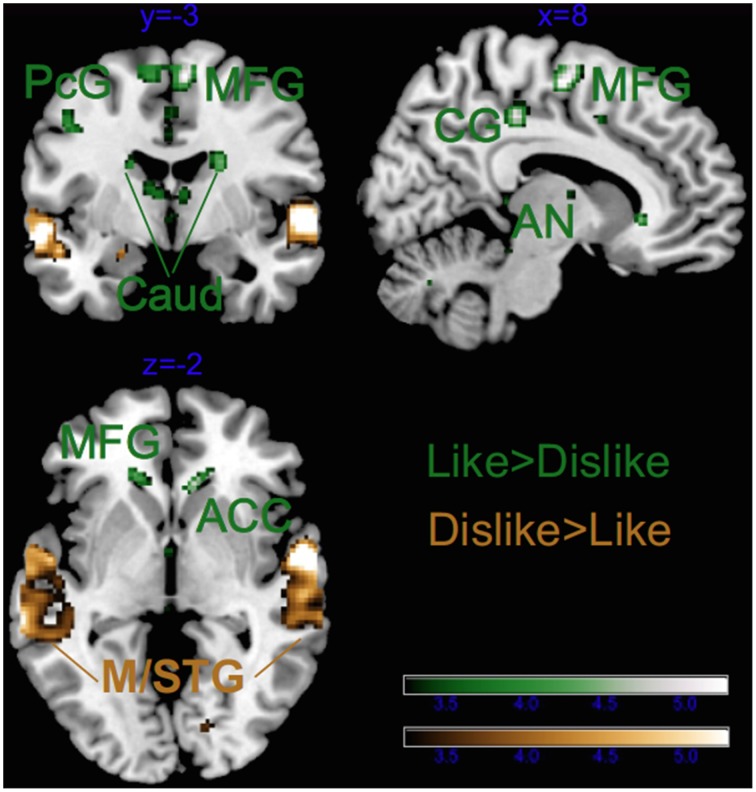
**Main effect of Liking for musicians only**. ACC, anterior cingulate cortex; Caud, caudate; CG, cingulate gyrus; STG, superior temporal gyrus; MTG, middle temporal gyrus; AN, anterior nucleus of the thalamus; PcG, precentral gyrus; and MFG, middle frontal gyrus. Activations were considered significant at *p* < 0.001, and cluster size *k*> 29 (as obtained with Monte Carlo permutations).

Figure 6 and Table 4, illustrate the results for the main effect of Emotion in musicians only. The contrast Happy > Sad revealed that only the bilateral auditory cortices were recruited whereas the opposite contrast (Sad > Happy) revealed no significant clusters. On the contrary, the Liked > Disliked music comparison widely recruited large clusters in the bilateral caudate nuclei (see Figure 7 and Table 4). Greater activations in the frontal lobe, with large right-hemispheric clusters including the medial frontal gyrus (including the orbitofrontal cortex) and the cingulate gyri, extending to the anterior cingulate were also obtained. The left frontal and adjacent parietal lobes were active with the medial frontal gyrus, the paracentral lobule, the inferior parietal lobule, and the precuneus. The right insula, the pulvinar thalamus, and the left declive of the cerebellum were also activated during listening to favorite music in musicians. On the other hand, Disliked > Liked music recruited only auditory areas in musicians, as evidenced from Figure 7 and Table 4.

**Table 4:** **Coordinates and statistical values of the full factorial analyses of fMRI responses performed separately for musicians**.

**MUSICIANS**								
**Region**	**L/R**	**BA**	***x***	***y***	***z***	***Z***	***k***	**Coordinates (x, y, z)**
**Main Effects**								
**LIKING**								
Middle/superior temporal gyrus	L	BA 21/22	−61	−8	−8	5.38	968	−62, −8, −10
Middle/superior temporal gyrus	R	BA 21/22	61	−6	−3	5.27	1058	62, −6, −4
Medial frontal gyrus	R/L	BA 6	6	−5	56	5.03	215	6, −8, 60
Cingulate gyrus	R	BA 31	10	−27	42	4.37	92	10, −30, 44
Precentral gyrus	L	BA 4	−48	−6	43	4.35	64	−48, −8, 46
Caudate body	L	N/A	−18	1	22	4.19	31	−18, 0, 24
Anterior cingulate	R	BA 24	10	27	−3	4.14	37	10, 28, −2
Caudate body	R	N/A	22	−1	22	3.99	49	22, −2, 24
Precuneus	L	BA 7	−12	−46	48	3.94	37	−12, −50, 50
Medial frontal gyrus	L	BA 10	−16	31	−3	3.76	29	−16, 32, −2
Cingulate gyrus	R	BA 32	2	10	40	3.64	34	2, 8, 44
Paracentral lobule	L	BA 31	−4	−27	46	3.60	39	−4, −30, 48
**EMOTION**								
Superior temporal gyrus	L	BA 22	−51	−4	−5	4.32	80	−52, −4, −6
***T*****-tests**								
**LIKE > DISLIkE**								
Medial frontal gyrus	R	BA 6	6	−5	56	5.16	287	6, −8, 60
Cingulate gyrus/paracentral lobule	R/L	BA 31	10	−27	42	4.52	202	10, −30, 44
Precentral gyrus	L	BA 4	−48	−6	43	4.50	89	−48, −8, 46
Caudate body	L	N/A	−18	1	22	4.34	46	−18, 0, 24
Anterior cingulate	R	BA 24	10	27	−3	4.30	59	10, 28, −2
Caudate body	R	N/A	22	−1	22	4.15	71	22, −2, 24
Precuneus	L	BA 7	−12	−46	48	4.10	53	−12, −50, 50
Medial frontal gyrus	L	BA 10	−16	31	−3	3.93	45	−16, 32, −2
Cingulate gyrus	R	BA 32/24	2	10	40	3.82	88	2, 8, 44
Pulvinar	R/L	N/A	6	−33	7	3.79	64	6, −34, 6
Caudate tail	L	N/A	−18	−22	20	3.74	31	−18, −24, 20
Declive	L	N/A	−8	−69	−17	3.71	39	−8, −70, −24
Inferior parietal lobule	L	BA 40	−50	−37	39	3.68	36	−50, −40,40
Insula	R	BA 13	44	4	5	3.49	32	44, 4, 6
**DISLIKE > LIKE**								
Middle/superior temporal gyrus	L	BA 21/22	−61	−8	−8	5.50	1300	−62, −8, −10
Middle/supeior temporal gyrus	R	BA 21/22	61	−6	−3	5.39	1298	62, −6, −4
**HAPPY > SAD**								
Superior temporal gyrus	L	BA 22	−51	−4	−5	4.47	138	−52, −4, −6
Superior temporal gyrus	R	BA 41	48	−27	9	3.82	41	48, −28, 8

*Clusters were considered significant at p < 0.001, and cluster size k > 29 (as obtained with Monte Carlo permutations). Coordinates are in MNI space. Only effects and interactions producing significant clusters are reported*.

#### Separate GLM for non-musicians

**Figure 8:**
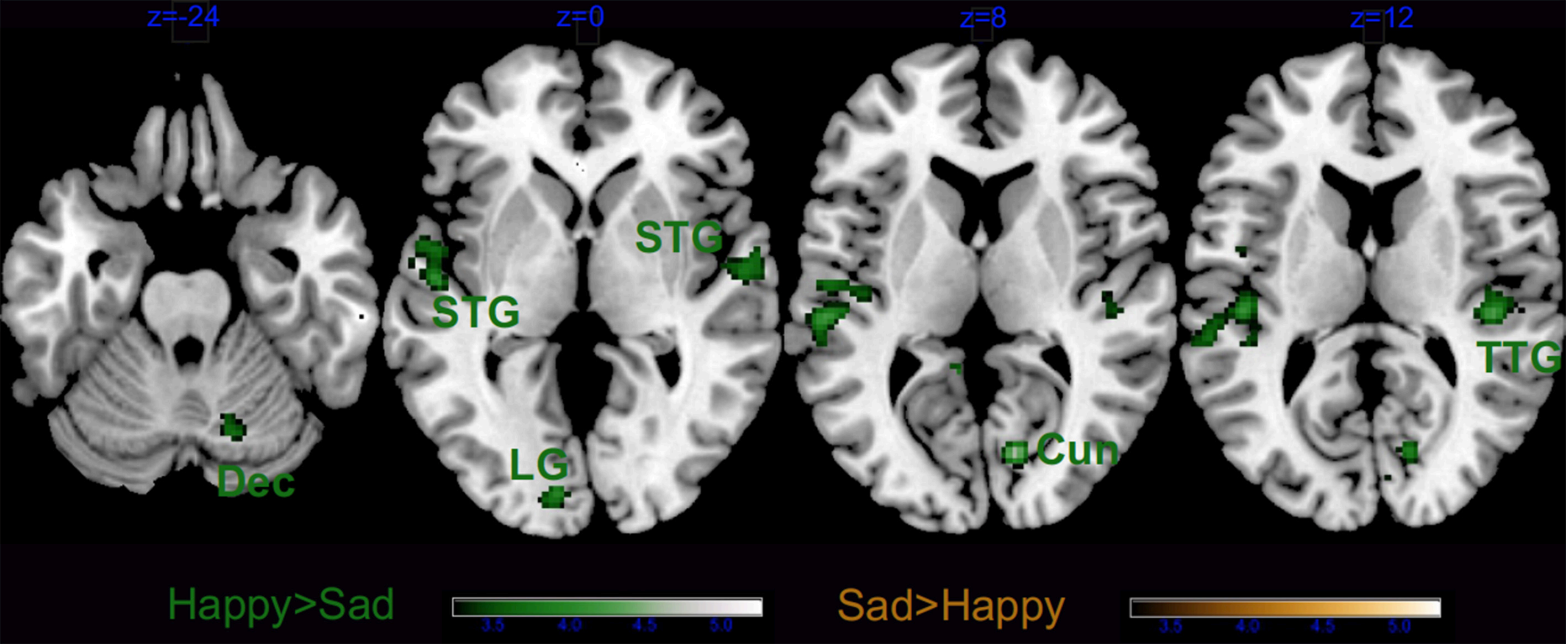
**Main effect of Emotion for non-musicians only**. Dec, cerebellar declive; STG, superior temporal gyrus; Cun, cuneus; TTG, transverse temporal gyrus; and LG, lingual gyrus. Activations were considered significant at *p* < 0.001, and cluster size *k* > 29 (as obtained with Monte Carlo permutations).

In non-musicians the results of the contrast Happy > Sad music revealed significant clusters in the right hemispheric auditory cortices, the left lingual gyrus, the right cuneus and the right declive of the cerebellum (Figure 8 and Table 5). As in musicians, no significant cluster to Sad > Happy music was found.

**Table 5:** **Coordinates and statistical values of the full factorial analyses of fMRI responses performed separately for non-musicians**.

**NON-MUSICIANS**								
**Region**	**L/R**	**BA**	***x***	***y***	***z***	***Z***	***k***	**MNI coord**
**Main Effects**								
**LIKING**								
Cerebellar tonsil	L	N/A	−8	−52	−34	4.84	80	−8, −52, −44
Medial frontal gyrus	R/L	BA 6	6	−3	63	4.58	148	6, −6, 68
Superior/middle temporal gyrus	R	BA 21/22	61	−12	−1	4.21	367	62, −12, −2
Culmen	R	N/A	20	−50	−21	3.95	113	20, −50, −28
Middle/superior temporal gyrus	L	BA 21	−59	−6	−5	3.92	175	−60, −6, −6
Cingulate gyrus	R	BA 31	4	−31	40	3.89	74	4, −34, 42
Precentral gyrus/superior temporal gyrus	L	BA 6/22	−61	3	13	3.85	32	−62, 2, 14
Culmen	R	N/A	4	−57	−19	3.68	51	4, −58, −26
Post-central gyrus	L	BA 3	−40	−21	45	3.63	69	−40, −24, 48
**EMOTION**								
Cuneus	R	BA 23	12	−73	11	4.08	76	12, −76, 8
Superior temporal gyrus	L	BA 22/41/42	−53	−12	−1	3.90	358	−54, −12, −2
Transverse temporal gyrus	R	BA 41	44	−23	12	3.77	65	44, −24, 12
Superior temporal gyrus	R	BA 22	57	−10	2	3.75	76	58, −10, 2
Lingual gyrus	L	BA 17	−12	−87	3	3.53	35	−12, −90, −2
**LIKING × EMOTION**								
Inferior frontal gyrus	R	BA 47	50	19	−8	4.28	55	50, 20, −8
***T*****-Tests**								
**LIKE > DISLIKE**								
Cerebellar tonsil	L	N/A	−8	−52	−34	4.97	99	−8, −52, −44
Medial/superior frontal gyrus	R/L	BA 6	6	−3	63	4.72	204	6, −6, 68
Uvula	L	N/A	−22	−65	−25	4.11	49	−22, −66, −34
Culmen	R	N/A	20	−50	−21	4.11	163	20, −50, −28
Cingulate gyrus	R	BA 31	4	−31	40	4.05	123	4, −34, 42
Precentral gyrus/superior temporal gyrus	L	BA 6/22	−61	3	13	4.02	52	−62, 2, 14
Culmen	R	N/A	4	−57	−19	3.85	71	4, −58, −26
Precuneus	R	BA 7	8	−56	47	3.84	31	8, −60, 48
Post-central gyrus	L	BA 3	−40	−21	45	3.81	123	−40, −24, 48
Middle frontal gyrus/precentral gyrus	L	BA 6	−24	−11	61	3.69	73	−24, −14, 66
**DISLIKE > LIKE**								
Superior/middle temporal gyrus	R	BA 21/22	61	−12	−1	4.37	470	62, −12, −2
Middle/superior temporal gyrus	L	BA 21	−59	−6	−5	4.08	348	−60, −6, −6
**HAPPY > SAD**								
Cuneus	R	BA 23	12	−73	11	0.277	116	12, −76, 8
Superior temporal gyrus	L	BA 22/41/42	−53	−12	−1	0.453	633	−54, −12, −2
Transverse temporal gyrus	R	BA 41/22	44	−23	12	0.603	114	44, −24, 12
Superior temporal gyrus	R	BA 22	57	−10	2	0.624	134	58, −10, 2
Lingual gyrus	L	BA 17	−12	−87	3	0.853	57	−12, −90, −2
Declive	R	N/A	16	−67	−17	0.969	43	16, −68, −24
**POSITIVE INTERACTION: LIKING × EMOTION (LH + DS>LS+DH)**								
Inferior frontal gyrus	R	BA 47	50	19	−8	4.44	85	50, 20, −8
**NEGATIVE INTERACTION: LIKING × EMOTION (LS + DH > LH + DS)**								
Paracentral lobule/superor parietal lobule	L	BA 5/7	−8	−40	59	4.17	51	−8, −44, 62
Superior temporal gyrus	R	BA 41	38	−35	5	3.70	36	38, −36, 4
Insula	R	BA 13	32	−24	21	3.59	30	32, −26, 22

*Clusters were considered significant at p < 0.001, and cluster size k> 29 (as obtained with Monte Carlo permutations). Coordinates are in MNI space. Only effects and interactions producing significant clusters are reported*.

**Figure 9:**
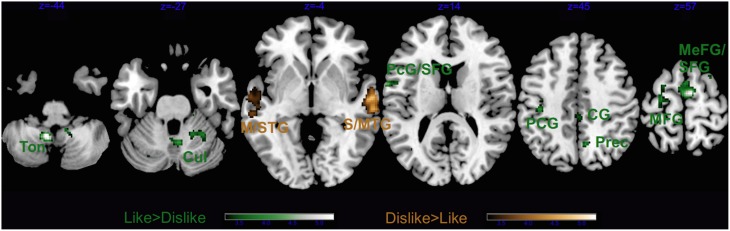
**Main effect of Liking for non-musicians only**. STG, superior temporal gyrus; MTG, middle temporal gyrus; PcG, precentral gyrus; SFG, superior frontal gyrus; PCG, post-central gyrus; CG, cingulate gyrus; MeFG, medial frontal gyrus; MFG, middle frontal gyrus; Prec, precuneus; Cul, cerebellar culmen; and Ton, cerebellar tonsil. Activations were considered significant at *p* < 0.001, and cluster size *k*> 29 (as obtained with Monte Carlo permutations).

The contrast Liked > Disliked music in non-musicians revealed larger brain activity bilaterally in the medial frontal gyrus, as well as the right cingulate gyrus, and the left superior temporal gyrus extending to the precentral and middle frontal gyri (Figure 9 and Table 5). The cerebellum was also recruited with the right culmen and the left tonsil and uvula. Similarly to musicians, Disliked > Liked music only activated the bilateral auditory cortices.

#### Correlations between acoustic features and BOLD signal

Pearson's correlation tests between the signal change extracted from the activated clusters for the main effect of Liking with the acoustic features that significantly differentiated the musical stimuli revealed a significant negative relation between the activity in the right declive of the cerebellum and the Dynamics-related features in the musical excerpts (*r* = −0.304, *p* = 0.001). The liking-related activity in the right-hemispheric declive also positively correlated with the Timbre-related features of the music (*r* = 0.335, *p* < 0.0001). Conversely, for the main effect of Emotion the Dynamics-related features negatively correlated with the neural activity in the right posterior cingulate gyrus (*r* = −0.241, *p* = 0.009) and to positively with the neural activity in the left superior temporal gyrus (*r* = 0.228, *p* = 0.01). However, these correlations did not survive Bonferroni correction.

## Discussion

This study provides two important contributions to the literatures on music emotions and expertise, respectively. First, by using an orthogonal design we succeeded in disentangling the brain structures responsible for the perception of sad and happy emotions in music from those related to liking and, hence, musical enjoyment. Second, we provided original evidence for functional differences in the limbic system between musicians and non-musicians, by showing enhanced liking-related activity in fronto-insular and cingulate areas (belonging to the salience processing network) in individuals who for years had been deeply enjoying music and were trained to convey emotions when performing.

### Neural correlates of music liking and disliking

In both groups, listening to their most liked music activated several deep subcortical and medial cortical structures of the brain belonging to neural networks controlling emotional and motivational experiences, that is, the limbic and paralimbic system (amygdala, parahippocampal gyrus, medial prefrontal cortex, anterior cingulate gyrus) and the reward circuit (caudate, medial prefrontal cortex, mediodorsal thalamus). The parahippocampal gyrus has previously been related to the discrimination of valence or pleasantness in music (Blood et al., 1999). The amygdala is instead supposed to have a broader alerting function as it processes the intensity, arousal potential and novelty of affective signals (e.g., Small et al., 2003; Sescousse et al., 2013; Frühholz et al., 2014). Indeed, in the musical domain, amygdalar activity has been reported for positive and negative emotions such as fear and joy (Eldar et al., 2007). Recruitment of the amygdala has also been associated with tonal novelty defined by the incongruity of chords ending a Western tonal cadence (Koelsch et al., 2008).

In the current study, the right amygdala was more active during listening to disliked music clips than during liked ones. Lateralized activity of the amygdala has been observed in a meta-analysis of studies for the visual modality (Fusar-Poli et al., 2009), but contrarily with our findings the left amygdala was associated with negative emotions while the right amygdala with positive ones. Our recent studies, though, confirm the reversed pattern of increased left amygdalar activity and connectivity during continuous listening to music judged as pleasant (Toiviainen et al., submitted; Bogert et al., submitted). As was also suggested in a recent review (Brattico, 2015), the discrepancy between findings obtained in visual and music studies might be related with the distinct functions of the features eliciting emotions in the two modalities; e.g., in vision amygdala is more strongly activated by low spatial frequency content (as compared to high frequency), to allow fast recognition of objects and potential danger such as that conveyed by fearful faces (Vuilleumier et al., 2003), whereas in audition fine spectrotemporal resolution is needed to discern potentially dangerous rough, dissonant signals, such as predator calls (Peretz, 2010).

In conjunction with the amygdala, the inferior frontal regions (Tillmann et al., 2006), the anterior superior temporal gyrus, and the basal ganglia (especially the head of the caudate; Seger et al., 2013) were activated in association with violations of different kinds of musical expectancies. Salimpoor et al. (2015) summarize that the posterior inferior frontal gyrus in the right hemisphere tracks expectancies in musical structure, such as how well chords fit to the preceding context according to the Western harmony rules of chord succession, caudate with temporal anticipation of a reward experience, whereas amygdala (and nucleus accumbens) activation is related to the emotional outcome of the expectancy processes. However, in our study emotion-related structures (in amygdala, parahippocampal gyrus, and caudate) co-activated with dorsomedial rather than inferolateral frontal regions, similarly to recent fMRI findings obtained with pleasant 10 sec excerpts from film soundtracks (Altenmüller et al., 2014). Metabolic activity in the premotor cortex, residing in the precentral frontal gyri, is related to somatomotor representations and imagery. Some of the clusters found active during listening to favorite music belong to the mirror neuron system, which are active not only during action execution but also during action observation (Rizzolatti et al., 1996; Hari et al., 1998). Some authors have hypothesized a role of the mirror neuron system in aesthetic enjoyment and emotion recognition for both the visual and auditory domains (Molnar-Szakacs and Overy, 2006; Gallese and Freedberg, 2007); however, there is scarce consensus in the field due to the yet insufficient evidence of mirror neurons in humans (Pascolo et al., 2010). Nevertheless, several studies using highly pleasurable music from different styles as stimuli also reported activity in premotor and supplementary motor areas (Blood and Zatorre, 2001; Salimpoor et al., 2013). An alternative explanation for neural activity in medial supplementary motor areas (BA 6, but not in primary motor cortex, M1) during affective music listening might be silent vocalization, previously documented in a study of imagined compared with overt singing of an Italian aria (Kleber et al., 2007).

In addition to limbic and motor structures, favorite music activated several thalamic clusters, including dorsomedial and pulvinar regions, which are considered as important value coding centers for reward experiences (Sescousse et al., 2013). In a meta-analysis of 87 neuroimaging studies, the dorsomedial thalamus was one of the structures consistently associated with different monetary, food and erotic rewards (Sescousse et al., 2013). Although it is rarely discussed in the reward literature, and even less in music studies, the dorsomedial thalamus is an important relay center between the basal ganglia and the prefrontal cortex. On the other hand, the pulvinar thalamus, which is connected with cingulate, associative parietal and premotor cortices (Cappe et al., 2009), was also found activated here. This striatal-thalamo-cortical loop, associated in the current study with liked music stimuli, might hence represent a bridge between reward signals in the ventral striatum and higher cognitive processes, such as motivation and goal-directed behavior, occurring in cingulate and frontal areas.

### Neural correlates of happiness and sadness in music

Listening to liked music (as opposed to disliked music) elicited neural activity in emotional and motivational structures of the brain, including deep subcortical centers controlling arousal and automatic nervous system activity, whereas listening to happy music (as contrasted to sad music) elicited activity in sensory areas, namely the bilateral primary and non-primary auditory cortices. This replicates previous findings using instrumental classical music (Mitterschiffthaler et al., 2007) and also music excerpts with lyrics from various styles (Brattico et al., 2011). Re-entry mechanisms from the nucleus accumbens into the auditory cortex, boosting the neural activity to happy-sounding, rewarding and motivating sounds, may be at the origin of this effect (Budinger and Scheich, 2009; Puschmann et al., 2013; Salimpoor et al., 2013). However, since also disliked music was included in the fMRI responses to happy musical excerpts, the above explanation holds only partly. An alternative, more likely explanation for the auditory cortex activity during happy music can be found in the acoustic characteristics of happy compared to sad music, such as brighter timbre, more staccato articulation, faster tempo and major tonality as found in the acoustic feature analysis of this study and in previous literature (Brattico et al., 2011) as well as in the specialization of the right and left auditory cortex for spectro-temporal attributes (Zatorre et al., 2002).

Posterior medial structures (lingual gyrus, cuneus, posterior cingulate gyrus) were previously found to be active during resting state (Fox et al., 2009) and also in association with processing of timbral features during continuous listening to music (Alluri et al., 2012). A neural dissociation between basic emotional processing and motivational processes in music has been hypothesized earlier (Vuilleumier and Trost, 2015) and hinted at from behavioral evidence (Fletcher et al., 2013), but thus far never demonstrated. In a previous behavioral study (Matthews et al., 2009), JS, a neurological patient suffering from auditory agnosia of undefined etiology showed chance level recognition of basic emotions (including their negative or positive content) in environmental sounds and musical pieces but preserved enjoyment of favorite music. In sum, our findings concerning the neural correlates of happiness and sadness in music replicate and also clarify previous findings by identifying the brain structures that are specifically activated by the discrete emotions, irrespectively of their hedonic value.

### Behavioral ratings relating liking with the intensity of discrete emotions perceived

These findings have been obtained by using music selected by the subjects themselves. This design was adopted for enhancing the potential of music to convey both basic and pleasurable emotions. Based on their behavioral ratings, subjects were successful in selecting music that was perceived as sad or happy. The excerpts extracted from the music, lasting 18 sec, were even able to induce happy and sad felt emotions coherent to expectations, and this was all the more true when the music was also preferred by the subjects, hence replicating behavioral findings (Kreutz et al., 2008; Salimpoor et al., 2009). The capacity of musical (or visual) stimuli to induce feelings in the participants —in other words, of being moved by them— has been considered as a pivotal psychological construct. According to recent accounts, the psychological state of being moved can alone explain the pleasure derived from negatively connoted aesthetic stimuli, such as sadness-inducing music or films or disgust-inducing pictures when displayed in an art context (Kreutz et al., 2008; Hanich et al., 2014; Wagner et al., 2014). Focusing on peak experiences in response to artworks, Konecni (2008) proposed an Aesthetic Trinity Theory including three separate aesthetic responses: “aesthetic awe,” defined at the rare, intense, highly memorable peak experience; the state of “being moved,” a more frequent and less intense experience; and thrills or chills, namely the physiological responses that in Konecni's opinion are more commonly occurring (although other reports found them to be rare; Grewe et al., 2009). In this regard, our data provide further evidence linking the psychological construct of being moved with stronger aesthetic responses to music, namely, higher emotion induction ratings for liked music. In an attempt to chronometrically organize the various processes and responses occurring during a musical aesthetic experience, Brattico et al. (2013) situated the aesthetic emotion of enjoyment in temporal succession after the induction and perception of discrete emotions from music and before liking, conceptualized as a conscious act of judgment of the preceding affective and cognitive reactions to the music heard.

### Controlling for familiarity

Our findings were obtained while controlling for familiarity of the musical excerpts. Based on post-experimental interviews and on the behavioral ratings, the excerpts belonging to the different experimental categories were all considered as familiar, corresponding to ratings exceeding the middle one in the scale, with slightly higher ratings of familiarity for the favorite sad music. While confident that the musical excerpts were all familiar to our subjects, even when disliked (and in several instances, those disliked pieces were very corny pop hits of the moment), we included the familiarity ratings from the listening test as individual covariates in the fMRI analysis. This allowed removing those additional effects in the reward areas and limbic system which by themselves would differentiate the liked from the disliked music or the happy from the sad one, simply based on previous listening exposure, as evidenced by a recent fMRI study (Pereira et al., 2011). Indeed, repeated exposure to musical excerpts increased ratings of emotional arousal, induced subjective reports of pleasure and also electrodermal activity in listeners (Salimpoor et al., 2009; van den Bosch et al., 2013).

### Sensory-motor brain responses in musicians and non-musicians

Complying with previous literature, the present neuroimaging findings revealed functional differences between the brains of musicians and non-musicians (Münte et al., 2002; Tervaniemi, 2012; Merrett et al., 2013; Reybrouck and Brattico, 2015; Schlaug, 2015). Particularly, musicians (as contrasted with non-musicians) showed an overall increased activity in somatomotor regions, including the precentral and post-central cerebral gyri and the cerebellar declive. Previously, neurophysiological studies demonstrated that the functional adaptations of somatomotor regions are dependent on the specific demands of instrumental practice. For instance, larger neurophysiological responses in the right somatosensory cortex, indexing more accurate representations of the fingers of the left hand in string players, were found as opposed to non-musicians, with the amount of response increase dependent on the years of training (Elbert et al., 1995). In experienced opera singers the increased functional activation dependent on the amount of singing practice has been found in the bilateral regions of the primary somatosensory cortex representing the articulatory vocal tract and larynx, and subcortically in the basal ganglia, thalamus and cerebellum (Kleber et al., 2013). A recent fMRI study also revealed less symmetric functional activation of somatomotor (and other) regions in string players as contrasted with keyboard players (Burunat et al., 2015). Long-term repeated activation of somatomotor cortex in musicians also results in permanent structural changes in the volume of the anatomical structures. Volumetric studies have repeatedly showed larger premotor cortex and cerebellum in (male) musicians as compared with non-musicians (Gärtner et al., 2013; Schlaug, 2015). The changes in right superior and middle frontal regions (along with the hippocampus) depend on the amount of training and are visible from the beginning of musical training and upward, as showed by a volumetric study contrasting musicians who started their training before 7 years of age with those who started later (Groussard et al., 2014), and in another study investigating children who studied music for just 15 months (Hyde et al., 2009). Moreover, a longer persistence with musical practice seems to be necessary for structural changes in the right insula, left posterior cingulate cortex, superior temporal areas, and right supplementary motor area (Groussard et al., 2014).

### Limbic system responses in musicians and non-musicians

Remarkably, while the analysis of the behavioral ratings from the listening test showed only marginal differences between experimental groups, partly replicating previous findings for pleasantness ratings of chords (Brattico et al., 2009) or classical music excerpts (Dellacherie et al., 2011), the fMRI responses clearly did differentiate the professional musicians from the controls. Those neural differences were only marginally influenced by the divergent sensory processing of the dynamics-, articulation-, and timbre-related acoustic features present in the music chosen by musicians and non-musicians, as shown by the few significant correlations between acoustic features and significant clusters of brain activity. Particularly, the brain activity in response to liked music, and hence to musical pleasure, localized in limbic areas including parts of the ventral striatum, the orbitofrontal cortex, the insula, and the parahippocampal gyrus, was more emphasized in musicians than non-musicians. In contrast, auditory, somatomotor and associative visual brain areas of non-musicians responded intensively to happy music. These findings, hinting at a modulatory effect of musical expertise on limbic system activity, resemble those obtained with fMRI measures of experts in compassion meditation (Lutz et al., 2008, 2009). This mental practice aims at learning to exercise empathy for other people's suffering. As a consequence of this intense and long-term practice in experts, the activity of the insula and anterior cingulate is enhanced compared with naïve subjects. In contrast, repeated negative affective experiences can also plastically shape regions of the limbic system: for instance, the basolateral amygdala is known to sensitize in post-traumatic stress disorder, namely in a condition dominated by constant fear (for a review, see Davidson and McEwen, 2012). Hence, the enhanced functionality of the limbic system observed here with musicians and previously with meditation experts can be considered as the adaptive, positive counterpart of the maladaptive limbic functionality, resulting from continuous activation of limbic areas by negative emotions.

The picture regarding modified affective functions in experts, as a result of exposure to emotionally loaded stimuli, is, however, somewhat more complex in an aesthetic domain such as music, in which training in understanding formal structures (such as tonal harmony for Western classical music) might surpass the training in recognizing and conveying emotions via music. Indeed, Cupchik and Laszlo (1992) identified a pleasure-based way of appreciating the arts, which is distinguishable from a cognitive-based way. Experts who have art knowledge that facilitates cognitive processing will refer to a more cognitive-based way to perceive the arts whereas those who are relatively naive will engage in a more emotional manner to appreciate. Results by Müller et al. (2010) obtained with electrophysiological recordings of music experts and non-experts rating music beauty confirm the prediction by Cupchik and Laszlo (1992), showing affective neural processes for non-experts only. Here, when two different kinds of affective processes are studied, we were able to identify the neural structures associated with musical emotions and liking differentiating musicians from non-musicians.

Indeed, in the music domain differences in the limbic functions, hinting at a putative affective neuroplasticity derived from music training, have been noticed only in sparse studies failing thus far to provide a coherent picture of the phenomenon. In a recent fMRI study (Chapin et al., 2010), right ventral striatal and anterior cingulate activity was enhanced in musicians during listening to expressive music (a prelude by Chopin played by a pianist) as compared to non-musicians. In a second study, musicians gave higher arousal ratings and showed increased activity of the right dorsolateral prefrontal cortex and right parietal regions in response to sad and fearful music, respectively, as opposed to non-musicians (Park et al., 2014). In contrast, happy music did not discriminate brain activity of musicians from that of non-musicians. Previously, James et al. (2008) noticed the increased role of the insula for visceral representation and bodily awareness in musicians (see similar findings obtained with singers by Kleber et al., 2007, 2013), which can be attributed to the fine-motoric demands of their daily repetitive practice (Zamorano et al., 2015). In a third recent study Luo et al. (2014) further reported increased functional connectivity between structures of the salience network (implicated in attentional and other high cognitive functions), namely the insula, anterior cingulate and temporoparietal junction, while relaxing with eyes closed, in musicians as compared with non-musicians. The scarcity of studies on differences in limbic functions and affective neuroplasticity in musicians might be ascribed to influential behavioral and brain findings putting forward a conception of musicians as listening to music in a cognitive, analytic way as opposed to the more emotional way of non-musicians (Bever and Chiarello, 1974; Hirshkowitz et al., 1978; Herholz et al., 2011). Added to this, neuroscience research has focused on perceptual and cognitive skills related to music processing and how they are modulated by musical expertise (Reybrouck and Brattico, 2015). Aesthetic processes including enjoyment and liking of music have been thus far much neglected in previous studies. Nevertheless, our findings encourage to studying putative neural adaptations of the limbic system in musicians. Remarkably, the areas that were found here more activated in musicians than non-musicians during affecting listening to music (insula, striatum, cingulate and the pulvinar thalamus) are known to be involved in visceral function and production of body maps. Habibi and Damasio (2015) proposed a link between musical pleasure and homeostatic regulation that receives some support from the present results with musicians' brains.

### Implications for recent accounts on musical aesthetic responses

The current experiment represents an initial step toward discerning the distinct psychological and neural processes comprising an aesthetic experience of music by finding separable neural substrates of liking or disliking music and perceiving music as happy or sad. While the neural correlates of happy and sad music were significantly related with the acoustic content of the music, as shown by significant correlations between acoustic feature parameters and brain clusters of activation, the neural substrates of musical enjoyment only marginally represented the divergent acoustic features contained in liked vs. disliked music since almost no correlations were found between acoustic features and brain clusters of activation.

While several authors in behavioral literature have noticed how the emotional content of music might diverge from its aesthetic enjoyment and preference, focusing mainly on the paradoxical phenomenon of liking or even preferring to listen to sad music, neural correlates of this dissociation have only been hypothesized in reviews rather than being empirically tested. For instance, Koelsch et al. (2015) proposed a distinction between pleasurable emotions (in our terminology enjoyment and conscious liking) activating the anterior hippocampus in communication with the ventral striatum and attachment-related, tender emotions (in this study, happy or joyful emotions from music) that are likely also controlled by the anterior hippocampus: “the experience of ‘having fun’ does not necessarily implicate joy, happiness” (p. 9).

According to our recent notions (Nieminen et al., 2011; Brattico and Pearce, 2013; Brattico et al., 2013; Reybrouck and Brattico, 2015), the perception of basic emotions in music occurs in neural structures spatially separate from motivational and evaluative processes such as aesthetic enjoyment, conscious liking and beauty (or other aesthetic) judgments. It also seems that these processes differ in their time course, basic emotions being processed earlier than motivational and evaluative processes. Our findings seem to be in line with these notions by indicating that enjoyment and conscious liking of a musical piece depends on an implicit appraisal of the general affective state induced by it (Brattico and Pearce, 2013; Brattico et al., 2013).

Future experiments should measure the temporal course of the emotional and evaluative processes studied here to provide the chronometrical succession of information stages during a musical aesthetics experience. Furthermore, information on the arousal level or intensity of the musical emotions related to the current stimulation was not directly obtained, although it can be partly inferred from ratings on the degree of felt emotions obtained in the listening test prior to fMRI scanning.

## Conclusions

The present findings demonstrate a neural dissociation between basic emotional responses to musical stimuli and evaluative pleasure-related processes on them, which are at the root of what Aristotle described as the “paradox of tragedy.” Furthermore, the study showed the modulation of these processes in the brains of musicians when opposed to non-musicians, with increased functionality of areas related to proprioception and salience detection, such as the insula and the anterior cingulate cortex, presumably derived from the long-term accustomization to music and expertise in expressing emotion through sounds. Longitudinal studies, though, are called for to demonstrate the causal relation between exposure to affective stimuli and adaptive changes in the limbic system.

### Conflict of interest statement

The authors declare that the research was conducted in the absence of any commercial or financial relationships that could be construed as a potential conflict of interest.

## References

[B1] Alluri V. Brattico E. Toiviainen P. Burunat I. Bogert B. Numminen J. (in press). Musical expertise modulates functional connectivity of limbic regions during continuous music listening. Psychomusicology.

[B2] Alluri V. Toiviainen P. (2010). Exploring perceptual and acoustical correlates of polyphonic timbre. Music Percept. 27, 223–241. 10.1525/mp.2010.27.3.223

[B3] Alluri V. Toiviainen P. Jääskeläinen I. P. Glerean E. Sams M. Brattico E. (2012). Large-scale brain networks emerge from dynamic processing of musical timbre, key and rhythm. Neuroimage 59, 3677–3689. 10.1016/j.neuroimage.2011.11.019 22116038

[B4] Altenmüller E. Siggel S. Mohammadi B. Samii A. Münte T. F. (2014). Play it again, Sam: brain correlates of emotional music recognition. Front. Psychol. 5:114. 10.3389/fpsyg.2014.00114 24634661 PMC3927073

[B5] Baumgartner T. Lutz K. Schmidt C. F. Jäncke L. (2006). The emotional power of music: how music enhances the feeling of affective pictures. Brain Res. 1075, 151–164. 10.1016/j.brainres.2005.12.065 16458860

[B6] Berridge K. C. Kringelbach M. L. (2008). Affective neuroscience of pleasure: reward in humans and animals. Psychopharmacology 199, 457–480. 10.1007/s00213-008-1099-6 18311558 PMC3004012

[B7] Bever T. G. Chiarello R. J. (1974). Cerebral dominance in musicians and non-musicians. Science 185, 537–539. 10.1126/science.185.4150.537 4841585

[B8] Blood A. J. Zatorre R. J. (2001). Intensely pleasurable responses to music correlate with activity in brain regions implicated in reward and emotion. Proc. Natl. Acad. Sci. U.S.A. 98, 11818–11823. 10.1073/pnas.191355898 11573015 PMC58814

[B9] Blood A. J. Zatorre R. J. Bermudez P. Evans A. C. (1999). Emotional responses to pleasant and unpleasant music correlate with activity in paralimbic brain regions. Nat. Neurosci. 2, 382–387. 10.1038/7299 10204547

[B10] Brattico E. (2015). From pleasure to liking and back: bottom-up and top-down neural routes to the aesthetic enjoyment of music, in Art, Aesthetics and the Brain, eds Huston J. P. Nadal M. Mora F. Agnati L. F. Conde C. J. C. (Oxford; New York, NY: Oxford University Press), 303–318.

[B11] Brattico E. Alluri V. Bogert B. Jacobsen T. Vartiainen N. Nieminen S.. (2011). A functional MRI study of happy and sad emotions in music with and without lyrics. Front. Psychol. 2:308. 10.3389/fpsyg.2011.00308 22144968 PMC3227856

[B12] Brattico E. Bogert B. Jacobsen T. (2013). Toward a neural chronometry for the aesthetic experience of music. Front. Psychol. 4:206. 10.3389/fpsyg.2013.00206 23641223 PMC3640187

[B13] Brattico E. Pallesen K. J. Varyagina O. Bailey C. Anourova I. Järvenpää M.. (2009). Neural discrimination of nonprototypical chords in music experts and laymen: an MEG study. J. Cogn. Neurosci. 21, 2230–2244. 10.1162/jocn.2008.21144 18855547

[B14] Brattico E. Pearce M. (2013). The neuroaesthetics of music. Psychol. Aesthet. Creat. Arts 7, 48–61. 10.1037/a0031624

[B15] Brown S. Martinez M. J. Parsons L. M. (2004). Passive music listening spontaneously engages limbic and paralimbic systems. Neuroreport 15, 2033–2037. 10.1097/00001756-200409150-00008 15486477

[B16] Brown R. M. Zatorre R. J. Penhune V. B. (2015). Expert music performance: cognitive, neural, and developmental bases. Prog. Brain Res. 217, 57–86. 10.1016/bs.pbr.2014.11.021 25725910

[B17] Budinger E. Scheich H. (2009). Anatomical connections suitable for the direct processing of neuronal information of different modalities via the rodent primary auditory cortex. Hear. Res. 258, 16–27. 10.1016/j.heares.2009.04.021 19446016

[B18] Burunat I. Brattico E. Puoliväli T. Ristaniemi T. Sams M. Toiviainen P. (2015). Action in perception: prominent visuo-motor functional symmetry in musicians during music listening. PLoS ONE 10:e0138238. 10.1371/journal.pone.0138238 26422790 PMC4589413

[B19] Cappe C. Morel A. Barone P. Rouiller E. M. (2009). The thalamocortical projection systems in primate: an anatomical support for multisensory and sensorimotor interplay. Cereb. Cortex 19, 2025–2037. 10.1093/cercor/bhn228 19150924 PMC2722423

[B20] Carlson E. Saarikallio S. Toiviainen P. Bogert B. Kliuchko M. Brattico E. (2015). Maladaptive and adaptive emotion regulation through music: a behavioral and neuroimaging study of males and females. Front. Hum. Neurosci. 9:466. 10.3389/fnhum.2015.00466 26379529 PMC4549560

[B21] Chapin H. Jantzen K. Kelso J. A. Steinberg F. Large E. (2010). Dynamic emotional and neural responses to music depend on performance expression and listener experience. PLoS ONE 5:e13812. 10.1371/journal.pone.0013812 21179549 PMC3002933

[B22] Cupchik G. C. Laszlo J. (1992). Emerging Visions of the Aesthetic Process: in Psychology, Semiology, and Philosophy. Cambridge: Cambridge University Press.

[B23] Damasio A. R. Grabowski T. J. Bechara A. Damasio H. Ponto L. L. Parvizi J.. (2000). Subcortical and cortical brain activity during the feeling of self-generated emotions. Nat. Neurosci. 3, 1049–1056. 10.1038/79871 11017179

[B24] Davidson J. Heartwood K. (1997). Songwriting for Beginners: An Easy Beginning. Van Nuys, CA: Alfred Publishing.

[B25] Davidson R. J. McEwen B. S. (2012). Social influences on neuroplasticity: stress and interventions to promote well-being. Nat. Neurosci. 15, 689–695. 10.1038/nn.3093 22534579 PMC3491815

[B26] Dellacherie D. Roy M. Hugueville L. Peretz I. Samson S. (2011). The effect of musical experience on emotional self-reports and psychophysiological responses to dissonance. Psychophysiology 48, 337–349. 10.1111/j.1469-8986.2010.01075.x 20701708

[B27] Eerola T. (2011). Are the emotions expressed in music genre-specific? An audio-based evaluation of datasets spanning classical, film, pop and mixed genres. J. New Music Res. 40, 349–366. 10.1080/09298215.2011.602195

[B28] Eerola T. Ferrer R. Alluri V. (2012). Timbre and affect dimensions: evidence from affect and similarity ratings and acoustic correlates of isolated instrument sounds. Mus. Percept. 30, 49–70. 10.1525/mp.2012.30.1.49

[B29] Ekman P. (1999). Basic emotions, in Handbook of Cognition and Emotion, eds Dalgleish T. Power M. (Sussex: John Wiley and Sons, Ltd), 45–60.

[B30] Elbert T. Pantev C. Wienbruch C. Rockstroh B. Taub E. (1995). Increased cortical representation of the fingers of the left hand in string players. Science 270, 305–307. 10.1126/science.270.5234.305 7569982

[B31] Eldar E. Ganor O. Admon R. Bleich A. Hendler T. (2007). Feeling the real world: limbic response to music depends on related content. Cereb. Cortex 17, 2828–2840. 10.1093/cercor/bhm011 17395609

[B32] Evans P. Schubert E. (2008). Relationships between expressed and felt emotions in music. Mus. Sci. 12, 75–99. 10.1177/102986490801200105

[B33] Fauvel B. Groussard M. Chetélat G. Fouquet M. Landeau B. Eustache F.. (2014). Morphological brain plasticity induced by musical expertise is accompanied by modulation of functional connectivity at rest. Neuroimage 90, 179–188. 10.1016/j.neuroimage.2013.12.065 24418502

[B34] Ferrer R. Eerola T. Vuoskoski J. K. (2013). Enhancing genre-based measures of music preference by user-defined liking and social tags. Psychol. Mus. 41, 499–518. 10.1177/0305735612440611

[B35] Fletcher P. D. Downey L. E. Witoonpanich P. Warren J. D. (2013). The brain basis of musicophilia: evidence from frontotemporal lobar degeneration. Front. Psychol. 4:347. 10.3389/fpsyg.2013.00347 23801975 PMC3689257

[B36] Flores-Gutiérrez E. O. Díaz J. L. Barrios F. A. Favila-Humara R. Guevara M. A. del Río-Portilla Y.. (2007). Metabolic and electric brain patterns during pleasant and unpleasant emotions induced by music masterpieces. Int. J. Psychophysiol. 65, 69–84. 10.1016/j.ijpsycho.2007.03.004 17466401

[B37] Fox M. D. Zhang D. Snyder A. Z. Raichle M. E. (2009). The global signal and observed anticorrelated resting state brain networks. J. Neurophysiol. 101, 3270–3283. 10.1152/jn.90777.2008 19339462 PMC2694109

[B38] Frühholz S. Trost W. Grandjean D. (2014). The role of the medial temporal limbic system in processing emotions in voice and music. Prog Neurobiol. 123, 1–17. 10.1016/j.pneurobio.2014.09.003 25291405

[B39] Fusar-Poli P. Placentino A. Carletti F. Allen P. Landi P. Abbamonte M.. (2009). Laterality effect on emotional faces processing: ALE meta-analysis of evidence. Neurosci. Lett. 452, 262–267. 10.1016/j.neulet.2009.01.065 19348735

[B40] Gallese V. Freedberg D. (2007). Mirror and canonical neurons are crucial elements in esthetic response. Trends Cogn. Sci. 11, 411 10.1016/j.tics.2007.07.006

[B41] Garrido S. Schubert E. (2013). Adaptive and maladaptive attraction to negative emotions in music. Mus. Sci. 17, 147–166. 10.1177/1029864913478305

[B42] Gärtner H. Minnerop M. Pieperhoff P. Schleicher A. Zilles K. Altenmüller E.. (2013). Brain morphometry shows effects of long-term musical practice in middle-aged keyboard players. Front. Psychol. 4:636. 10.3389/fpsyg.2013.00636 24069009 PMC3779931

[B43] Gosselin N. Peretz I. Johnsen E. Adolphs R. (2007). Amygdala damage impairs emotion recognition from music. Neuropsychologia 45, 236–244. 10.1016/j.neuropsychologia.2006.07.012 16970965

[B44] Grewe O. Kopiez R. Altenmüller E. (2009). The chill parameter: goose bumps and shivers as promising measures in emotion research. Mus. Percept. 27, 61–74. 10.1525/mp.2009.27.1.61

[B45] Groussard M. Viader F. Landeau B. Desgranges B. Eustache F. Platel H. (2014). The effects of musical practice on structural plasticity: the dynamics of grey matter changes. Brain Cogn. 90, 174–180 10.1016/j.bandc.2014.06.013 25127369

[B46] Habibi A. Damasio A. (2015). Music, feelings, and the human brain. Psychomusicology 24, 92–102. 10.1037/pmu0000033

[B47] Hanich J. Wagner V. Shah M. Jacobsen T. Menninghaus W. (2014). Why we like to watch sad films. The pleasure of being moved in aesthetic experiences. Psychol. Aesthet. Creat. Arts 8, 130–143. 10.1037/a0035690

[B48] Hari R. Forss N. Avikainen S. Kirveskari E. Salenius S. Rizzolatti G. (1998). Activation of human primary motor cortex during action observation: a neuromagnetic study. Proc. Natl. Acad. Sci. U.S.A. 95, 15061–15065. 10.1073/pnas.95.25.15061 9844015 PMC24575

[B49] Herholz S. C. Boh B. Pantev C. (2011). Musical training modulates encoding of higher-order regularities in the auditory cortex. Eur. J. Neurosci. 34, 524–529. 10.1111/j.1460-9568.2011.07775.x 21801242

[B50] Hirshkowitz M. Earle J. Paley B. (1978). EEG alpha asymmetry in musicians and non-musicians: a study of hemispheric specialization. Neuropsychologia 16, 125–128. 10.1016/0028-3932(78)90052-0 634458

[B51] Hyde K. L. Lerch J. Norton A. Forgeard M. Winner E. Evans A. C.. (2009). The effects of musical training on structural brain development: a longitudinal study. Ann. N.Y. Acad. Sci. 1169, 182–186 10.1111/j.1749-6632.2009.04852.x 19673777

[B52] James C. E. Britz J. Vuilleumier P. Hauert C. A. Michel C. M. (2008). Early neuronal responses in right limbic structures mediate harmony incongruity processing in musical experts. Neuroimage 42, 1597–1608. 10.1016/j.neuroimage.2008.06.025 18640279

[B53] Jäncke L. Kühnis J. Rogenmoser L. Elmer S. (2015). Time course of EEG oscillations during repeated listening of a well-known aria. Front. Hum. Neurosci. 9:401. 10.3389/fnhum.2015.00401 26257624 PMC4507057

[B54] Juslin P. N. Laukka P. (2004). Expression, perception, and induction of musical emotions: a review and a questionnaire study of everyday listening. J. New Mus. Res. 33, 217–238. 10.1080/0929821042000317813

[B55] Kawakami A. Furukawa K. Katahira K. Okanoya K. (2013). Sad music induces pleasant emotion. Front. Psychol. 4:311. 10.3389/fpsyg.2013.00311 23785342 PMC3682130

[B56] Kawakami A. Furukawa K. Okanoya K. (2014). Music evokes vicarious emotions in listeners. Front. Psychol. 5:431. 10.3389/fpsyg.2014.00431 24910621 PMC4038858

[B57] Khalfa S. Schön D. Anton J. L. Liégeois-Chauvel C. (2005). Brain regions involved in the recognition of happiness and sadness in music. Neuroreport 16, 1981–1984. 10.1097/00001756-200512190-00002 16317338

[B58] Kirk U. Skov M. Christensen M. S. Nygaard N. (2009). Brain correlates of aesthetic expertise: a parametric fMRI study. Brain Cogn. 69, 306–315. 10.1016/j.bandc.2008.08.004 18783864

[B59] Kleber B. Birbaumer N. Veit R. Trevorrow T. Lotze M. (2007). Overt and imagined singing of an Italian aria. Neuroimage 36, 889–900. 10.1016/j.neuroimage.2007.02.053 17478107

[B60] Kleber B. Zeitouni A. G. Friberg A. Zatorre R. J. (2013). Experience-dependent modulation of feedback integration during singing: role of the right anterior insula. J. Neurosci. 33, 6070–6080. 10.1523/JNEUROSCI.4418-12.2013 23554488 PMC6618920

[B61] Koelsch S. (2010). Towards a neural basis of music-evoked emotions. Trends Cogn. Sci. 14, 131–137. 10.1016/j.tics.2010.01.002 20153242

[B62] Koelsch S. (2014). Brain correlates of music-evoked emotions. Nat. Rev. Neurosci. 15, 170–180. 10.1038/nrn3666 24552785

[B63] Koelsch S. Fritz T. Schlaug G. (2008). Amygdala activity can be modulated by unexpected chord functions during music listening. Neuroreport 19, 1815–1819. 10.1097/WNR.0b013e32831a8722 19050462

[B64] Koelsch S. Fritz T. von Cramon D. Y. Muller K. Friederici A. D. (2006). Investigating emotion with music: an fMRI study. Hum. Brain Mapp. 27, 239–250. 10.1002/hbm.20180 16078183 PMC6871371

[B65] Koelsch S. Jacobs A. M. Menninghaus W. Liebal K. Klann-Delius G. von Scheve C.. (2015). The quartet theory of human emotions: an integrative and neurofunctional model. Phys. Life Rev. 13, 1–27. 10.1016/j.plrev.2015.03.001 25891321

[B66] Konecni V. J. (2008). Does music induce emotion? A theoretical and methodological analysis. Psychol. Aesthet. Creat. Arts 2, 115–129. 10.1037/1931-3896.2.2.115

[B67] Kreutz G. Ott U. Teichmann D. Osawa P. Vaitl D. (2008). Using music to induce emotions: influences of musical preference and absorption. Psychol. Mus. 36, 101–126. 10.1177/0305735607082623

[B68] Kringelbach M. L. (2005). The human orbitofrontal cortex: linking reward to hedonic experience. Nat. Neurosci. Rev. 6, 691–702. 10.1038/nrn1747 16136173

[B69] Krumhansl C. L. (1997). An exploratory study of musical emotions and psychophysiology. Can. J. Exp. Psychol. 51, 336–353. 10.1037/1196-1961.51.4.336 9606949

[B70] Lartillot O. Toiviainen P. (2007). A Matlab toolbox for musical feature extraction from audio, in Paper Presented at the 10th Int. Conference on Digital Audio Effects (DAFx-07) (Bordeaux).

[B71] LeBlanc A. Sims W. L. Silvola C. Obert M. (1996). Music style preferences of different age listeners. J. Res. Mus. Educ. 44, 49–59. 10.2307/3345413

[B72] Lindquist K. A. Wager T. D. Kober H. Bliss-Moreau E. Barrett L. F. (2012). The brain basis of emotion: a meta-analytic review. Behav. Brain Sci. 35, 121–143. 10.1017/S0140525X11000446 22617651 PMC4329228

[B73] Luo C. Tu S. Peng Y. Gao S. Li J. Dong L.. (2014). Long-term effects of musical training and functional plasticity in salience system. Neural Plasticity 2014:180138. 10.1155/2014/180138 25478236 PMC4247966

[B74] Lutz A. Brefczynski-Lewis J. Johnstone T. Davidson R. J. (2008). Regulation of the neural circuitry of emotion by compassion meditation: effects of meditative expertise. PLoS ONE 3:e1897. 10.1371/journal.pone.0001897 18365029 PMC2267490

[B75] Lutz A. Greischar L. L. Perlman D. M. Davidson R. J. (2009). BOLD signal in insula is differentially related to cardiac function during compassion meditation in experts vs. novices. Neuroimage 47, 1038–1046. 10.1016/j.neuroimage.2009.04.081 19426817 PMC2854658

[B76] Matthews B. R. Chang C. C. De May M. Engstrom J. Miller B. L. (2009). Pleasurable emotional response to music: a case of neurodegenerative generalized auditory agnosia. Neurocase 15, 248–259. 10.1080/13554790802632934 19253088 PMC2829118

[B77] Menon V. Levitin D. J. (2005). The rewards of music listening: response and physiological connectivity of the mesolimbic system. Neuroimage 28, 175–184. 10.1016/j.neuroimage.2005.05.053 16023376

[B78] Merrett D. L. Peretz I. Wilson S. J. (2013). Moderating variables of music training-induced neuroplasticity: a review and discussion. Front. Psychol. 4:606. 10.3389/fpsyg.2013.00606 24058353 PMC3766835

[B79] Mikutta C. Altorfer A. Strik W. Koenig T. (2012). Emotions, arousal, and frontal alpha rhythm asymmetry during Beethoven's 5th symphony. Brain Topogr. 25, 423–430. 10.1007/s10548-012-0227-0 22534936

[B80] Mikutta C. A. Maissen G. Altorfer A. Strik W. Koenig T. (2014). Professional musicians listen differently to music. Neuroscience 268, 102–111. 10.1016/j.neuroscience.2014.03.007 24637097

[B81] Mitterschiffthaler M. T. Fu C. H. Dalton J. A. Andrew C. M. Williams S. C. (2007). A functional MRI study of happy and sad affective states induced by classical music. Hum. Brain Mapp. 28, 1150–1162. 10.1002/hbm.20337 17290372 PMC6871455

[B82] Molnar-Szakacs I. Overy K. (2006). Music and mirror neurons: from motion to ‘e’motion. Soc. Cogn. Affect. Neurosci. 1, 235–241. 10.1093/scan/nsl029 18985111 PMC2555420

[B83] Müller M. Höfel L. Brattico E. Jacobsen T. (2010). Aesthetic judgments of music in experts and laypersons—an ERP study. Int. J. Psychophysiol. 76, 40–51. 10.1016/j.ijpsycho.2010.02.002 20153786

[B84] Münte T. F. Altenmüller E. Jäncke L. (2002). The musicians' brain as a model of neuroplasticity. Nat. Rev. Neurosci. 3, 473–478. 10.1038/nrn843 12042882

[B85] Nieminen S. Istok E. Brattico E. Tervaniemi M. (2012). The development of the aesthetic experience of music: preference, emotions, and beauty. Mus. Sci. 16, 372–391. 10.1177/1029864912450454

[B86] Nieminen S. Istók E. Brattico E. Tervaniemi M. Huotilainen M. (2011). The development of aesthetic responses to music and their underlying neural and psychological mechanisms. Cortex 47, 1138–1146. 10.1016/j.cortex.2011.05.008 21665202

[B87] Pallesen K. J. Brattico E. Bailey C. Korvenoja A. Koivisto J. Gjedde A.. (2005). Emotion processing of major, minor, and dissonant chords: a functional magnetic resonance imaging study. Ann. N.Y. Acad. Sci. 1060, 450–453 10.1196/annals.1360.047 16597801

[B88] Pantev C. Paraskevopoulos E. Kuchenbuch A. Lu Y. Herholz S. C. (2015). Musical expertise is related to neuroplastic changes of multisensory nature within the auditory cortex. Eur. J. Neurosci. 41, 709–717. 10.1111/ejn.12788 25728187

[B89] Park M. Gutyrchik E. Bao Y. Zaytseva Y. Carl P. Welker L.. (2014). Differences between musicians and non-musicians in neuro-affective processing of sadness and fear expressed in music. Neurosci. Lett. 566, 120–124. 10.1016/j.neulet.2014.02.041 24582901

[B90] Park M. Hennig-Fast K. Bao Y. Carl P. Pöppel E. Welker L.. (2013). Personality traits modulate neural responses to emotions expressed in music. Brain Res. 1523, 68–76. 10.1016/j.brainres.2013.05.042 23732338

[B91] Pascolo P. B. Budai R. Rossi R. (2010). Critical review of the research leading to the mirror neuron paradigm - biomed 2010. Biomed. Sci. Instrum. 46, 422–427. 20467117

[B92] Pereira C. S. Teixeira J. Figueiredo P. Xavier J. Castro S. L. Brattico E. (2011). Music and emotions in the brain: familiarity matters. PLoS ONE 6:e27241. 10.1371/journal.pone.0027241 22110619 PMC3217963

[B93] Peretz I. (2010). Towards a neurobiology of musical emotions, in Handbook of Music and Emotion—Theory, Research, Applications, eds Juslin P. N. Sloboda J. A. (Oxford; New York, NY: Oxford University Press), 99–126.

[B94] Peretz I. Gagnon L. Bouchard B. (1998). Music and emotion: perceptual determinants, immediacy, and isolation after brain damage. Cognition 68, 111–141. 10.1016/S0010-0277(98)00043-2 9818509

[B95] Puschmann S. Brechmann A. Thiel C. M. (2013). Learning-dependent plasticity in human auditory cortex during appetitive operant conditioning. Hum. Brain Mapp. 34, 2841–2851. 10.1002/hbm.22107 22610479 PMC6870389

[B96] Reybrouck M. Brattico E. (2015). Neuroplasticity beyond sounds: neural adaptations following long-term musical aesthetic experiences. Brain Sci. 5, 69–91. 10.3390/brainsci5010069 25807006 PMC4390792

[B97] Rizzolatti G. Fadiga L. Gallese V. Fogassi L. (1996). Premotor cortex and the recognition of motor actions. Cogn. Brain Res. 3, 131–141. 10.1016/0926-6410(95)00038-0 8713554

[B98] Saarikallio S. (2008). Music in mood regulation: initial scale development. Mus. Sci. 12, 291–309. 10.1177/102986490801200206

[B99] Saarikallio S. Nieminen S. Brattico E. (2013). Affective reactions to musical stimuli reflect emotional use of music in everyday life. Mus. Sci. 17, 27–39. 10.1177/1029864912462381

[B100] Sachs M. E. Damasio A. Habibi A. (2015). The pleasures of sad music: a systematic review. Front. Hum. Neurosci. 9:404. 10.3389/fnhum.2015.00404 26257625 PMC4513245

[B101] Salimpoor V. N. Benovoy M. Larcher K. Dagher A. Zatorre R. J. (2011). Anatomically distinct dopamine release during anticipation and experience of peak emotion to music. Nat. Neurosci. 14, 257–262. 10.1038/nn.2726 21217764

[B102] Salimpoor V. N. Benovoy M. Longo G. Cooperstock J. R. Zatorre R. J. (2009). The rewarding aspects of music listening are related to degree of emotional arousal. PLoS ONE 4:e7487. 10.1371/journal.pone.0007487 19834599 PMC2759002

[B103] Salimpoor V. N. van den Bosch I. Kovacevic N. McIntosh A. R. Dagher A. Zatorre R. J. (2013). Interactions between the nucleus accumbens and auditory cortices predict music reward value. Science 340, 216–219. 10.1126/science.1231059 23580531

[B104] Salimpoor V. N. Zald D. H. Zatorre R. J. Dagher A. McIntosh A. R. (2015). Predictions and the brain: how musical sounds become rewarding. Trends Cogn. Sci. 19, 86–91. 10.1016/j.tics.2014.12.001 25534332

[B105] Scherer K. R. (2004). Which emotions can be induced by music? What are the underlying mechanisms? And how can we measure them? J. New Mus. Res. 33, 239–251. 10.1080/0929821042000317822

[B106] Schlaug G. (2015). Musicians and music making as a model for the study of brain plasticity. Prog. Brain Res. 217, 37–55. 10.1016/bs.pbr.2014.11.020 25725909 PMC4430083

[B107] Schubert E. (1996). Enjoyment of negative emotions in music: an associative network explanation. Psychol. Mus. 24, 18–28. 10.1177/0305735696241003

[B108] Seger C. A. Spiering B. J. Sares A. G. Quraini S. I. Alpeter C. David J.. (2013). Corticostriatal contributions to musical expectancy perception. J. Cogn. Neurosci. 25, 1062–1077. 10.1162/jocn_a_00371 23410032

[B109] Sescousse G. Caldú X. Segura B. Dreher J. C. (2013). Processing of primary and secondary rewards: a quantitative meta-analysis and review of human functional neuroimaging studies. Neurosci. Biobehav. Rev. 37, 681–696. 10.1016/j.neubiorev.2013.02.002 23415703

[B110] Silvia P. J. (2005). Emotional responses to art: from collation and arousal to cognition and emotion. Rev. Gen. Psychol. 9, 342–357. 10.1037/1089-2680.9.4.342

[B111] Sloboda J. A. (1992). Empirical studies of emotional response to music, in Cognitive Bases of Musical Communication, eds Jones M. R. Holleran S. (Washington, DC: American Psychological Association), 33–46.

[B112] Small D. M. Gregory M. D. Mak Y. E. Gitelman D. Mesulam M. M. Parrish T. (2003). Dissociation of neural representation of intensity and affective valuation in human gustation. Neuron 39, 701–711. 10.1016/S0896-6273(03)00467-7 12925283

[B113] Suzuki M. Okamura N. Kawachi Y. Tashiro M. Arao H. Hoshishiba T.. (2008). Discrete cortical regions associated with the musical beauty of major and minor chords. Cogn. Affect. Behav. Neurosci. 8, 126–131. 10.3758/CABN.8.2.126 18589503

[B114] Taruffi L. Koelsch S. (2014). The paradox of music-evoked sadness: an online survey. PLoS ONE 9:e110490. 10.1371/journal.pone.0110490 25330315 PMC4203803

[B115] Tervaniemi M. (2012). Musicianship—how and where in the brain?, in Musical Imaginations: Multidisciplinary Perspectives on Creativity, Performance and Perception, eds Hargreaves D. Miell D. MacDonald R. A. R. (Oxford; New York, NY: Oxford University Press), 285–295.

[B116] Tillmann B. Koelsch S. Escoffier N. Bigand E. Lalitte P. Friederici A. D.. (2006). Cognitive priming in sung and instrumental music: activation of inferior frontal cortex. Neuroimage 31, 1771–1782. 10.1016/j.neuroimage.2006.02.028 16624581

[B117] Trost W. Frühholz S. Cochrane T. Cojan Y. Vuilleumier P. (2015). Temporal dynamics of musical emotions examined through intersubject synchrony of brain activity. Soc. Cogn. Affect. Neurosci. 10, 1705–1721. 10.1093/scan/nsv060 25994970 PMC4666110

[B118] van den Bosch I. Salimpoor V. N. Zatorre R. J. (2013). Familiarity mediates the relationship between emotional arousal and pleasure during music listening. Front. Hum. Neurosci. 7:534. 10.3389/fnhum.2013.00534 24046738 PMC3763198

[B119] Van den Tol A. J. M. Edwards J. (2013). Exploring a rationale for choosing to listen to sad music when feeling sad. Psychol. Mus. 41, 440–465. 10.1177/0305735611430433

[B120] Vuilleumier P. Armony J. L. Driver J. Dolan R. J. (2003). Distinct spatial frequency sensitivities for processing faces and emotional expressions. Nat. Neurosci. 6, 624–631. 10.1038/nn1057 12740580

[B121] Vuilleumier P. Trost W. (2015). Music and emotions: from enchantment to entrainment. Neurosci. Mus. V Cogn. Stimul. Rehabilit. 1337, 212–222. 10.1111/nyas.12676 25773637

[B122] Vuoskoski J. K. Eerola T. (2011). The role of mood and personality in the perception of emotions represented by music. Cortex 47, 1099–1106. 10.1016/j.cortex.2011.04.011 21612773

[B123] Vuoskoski J. K. Eerola T. (2012). Can sad music really make you sad? Indirect measures of affective states induced by music and autobiographical memories. Psychol. Aesthet. Creat. Arts 6, 204–213. 10.1037/a0026937

[B124] Vuoskoski J. K. Thompson W. F. McIlwain D. Eerola T. (2012). Who enjoys listening to sad music and why? Mus. Percept. 29, 311–317. 10.1525/mp.2012.29.3.311

[B125] Wagner V. Menninghaus W. Hanich J. Jacobsen T. (2014). Art schema effects on affective experience: the case of disgusting images. Psychol. Aesthet. Creat. Arts 8, 120–129. 10.1037/a0036126

[B126] Zamorano A. M. Riquelme I. Kleber B. Altenmüller E. Hatem S. M. Montoya P. (2015). Pain sensitivity and tactile spatial acuity are altered in healthy musicians as in chronic pain patients. Front. Hum. Neurosci. 8:e1016. 10.3389/fnhum.2014.01016 25610384 PMC4285087

[B127] Zatorre R. J. Belin P. Penhune V. B. (2002). Structure and function of auditory cortex: music and speech. Trends Cogn. Sci. 6, 37–46. 10.1016/S1364-6613(00)01816-7 11849614

[B128] Zatorre R. J. Salimpoor V. N. (2013). From perception to pleasure: music and its neural substrates. Proc. Natl. Acad. Sci. U.S.A. 110, 10430–10437. 10.1073/pnas.1301228110 23754373 PMC3690607

[B129] Zentner M. Grandjean D. Scherer K. R. (2008). Emotions evoked by the sound of music: characterization, classification, and measurement. Emotion 8, 494–521. 10.1037/1528-3542.8.4.494 18729581

